# Powders of Diamond Nanoparticles as a Promising Material for Reflectors of Very Cold and Cold Neutrons

**DOI:** 10.3390/nano14040387

**Published:** 2024-02-19

**Authors:** Egor Lychagin, Marc Dubois, Valery Nesvizhevsky

**Affiliations:** 1 Frank Laboratory of Neutron Physics, Joint Institute for Nuclear Research, 141980 Dubna, Russia; 2 Clermont Auvergne INP, Université Clermont Auvergne, 63178 Aubière, France; 3 NPP/DS, Institut Max von Laue-Paul Langevin, 38042 Grenoble, France

**Keywords:** nanodiamonds, fluorination, very cold neutrons, cold neutrons, neutron reflectors

## Abstract

More than 15 years ago, the study of nanodiamond (ND) powders as a material for designing reflectors of very cold neutrons (VCNs) and cold neutrons (CNs) began. Such reflectors can significantly increase the efficiency of using such neutrons and expand the scope of their application for solving applied and fundamental problems. This review considers the principle of operation of VCN and CN reflectors based on ND powders and their advantages. Information is presented on the performed experimental and theoretical studies of the effect of the size, structure, and composition of NDs on the efficiency of reflectors. Methods of chemical and mechanical treatments of powders in order to modify their chemical composition and structure are discussed. The aim is to avoid, or at least to decrease, the neutron inelastic scatterers and absorbers (mainly hydrogen atoms but also metallic impurities and nitrogen) as well as to enhance coherent elastic scattering (to destroy ND clusters and sp^2^ carbon shells on the ND surface that result from the preparation of NDs). Issues requiring further study are identified. They include deeper purification of NDs from impurities that can be activated in high radiation fluxes, the stability of NDs in high radiation fluxes, and upscaling methods for producing larger quantities of ND powders. Possible ways of solving these problems are proposed.

## 1. Introduction

Due to their properties, such as their low toxicity, stable fluorescence, easy fuctionalization (modifiability), biocompatibility, and high hardness, nanodiamonds (NDs) [1,2,3,4] are considered a promising material for a number of applications in tribology, the modification of material properties, drug delivery, cosmetology, bioimaging, biosensorics, energy storage, catalysis, electronic applications, quantum engineering, etc. (see, for example, reviews [5,6] and the references therein). Most of these applications are still under development. NDs are most widely used as abrasive additives to lubricants and in the design of wear-resistant coatings [7]. There is an extensive literature and many reviews devoted to the study of various properties of NDs (crystal formation process, crystal lattice properties, their shape, structure of agglomerates, surface chemistry, various methods of surface modification, etc. (see, for example, refs. [5,8,9,10,11,12,13])).

To study the properties of NDs, a wide range of both chemical and physical methods are used. Scientists working in different areas of research, when determining the properties of NDs, often use “standard” (for them) research methods, the results of which can differ significantly. For example, the crystal lattice parameters determined from X-ray diffraction (XRD) [14] and neutron diffraction [15] usually agree well with each other, while the characteristic ND sizes determined from the results of small-angle neutron scattering (SANS) and small-angle X-ray scattering (SAXS) [16] usually give much smaller values than those from the results of dynamic light scattering (DLS) [17], since the latter are highly sensitive to ND clusters [18,19,20]. The emergence of a potentially new field of application for NDs and the corresponding shift in the focus of research often enriches our understanding of NDs with new data.

More than 15 years ago, the study of ND powders as a promising material for designing reflectors for very cold neutrons (VCNs) and cold neutrons (CNs) began. Neutron science and technology have played a huge role for almost a century and continue to be important today [21]. Neutron reflectors [22,23,24] are of great importance. Their main purpose is to redirect neutron fluxes to the right place and thus reduce neutron losses. They increase the performance of neutron sources and transport systems in an economical and efficient manner. Existing reflectors are inefficient in the VCN range. The design of reflectors of such neutrons based on NDs can significantly increase VCN/CN fluxes and expand the scope of such neutrons for solving applied and fundamental problems. In particular, some of these topics were recently mentioned when discussing particle physics at the European Spallation Source in Lund, Sweden [25,26,27,28,29,30,31,32,33].

This review considers the principle of the operation of reflectors based on NDs and the advantages of ND powders as VCN and CN reflectors. Information is presented on the experimental and theoretical studies performed to date on the effect of the size, structure, and composition of NDs on the efficiency of reflectors as well as on the methods of chemical and mechanical treatment of powders developed for these studies in order to change their chemical composition and structure. A range of issues requiring further study, the existing problems (limiting the possibility of application), and the possible ways to solve them are indicated.

To date, there are several technologies for producing diamond crystals in the nanometer size range in the form of particles. Various technologies for splitting larger crystals do not allow for the production of NDs smaller than 20–30 nm [34,35]. There are several technologies for the synthesis of NDs with a characteristic size of ∼5 nm. These methods include detonation synthesis [1], laser synthesis [36,37,38,39], ultrasound synthesis [40], and chemical vapor deposition [41,42,43]. All of these methods have different combinations of advantages and disadvantages.

In this review paper, we primarily deal with NDs synthesised via detonation, or detonation nanodiamonds (DNDs). The technology for producing DNDs was first developed more than 60 years ago [4]; however, even now, only this technology allows for the production of ND powders on an industrial scale.

## 2. Conventional Neutron Reflectors and Their Applications

The cores of nuclear reactors, irradiation cavities, and neutron moderators at any neutron source are surrounded by neutron reflectors. For nuclear power reactors, they return neutrons to the core, which allows for a significant reduction in the amount of nuclear fuel (usually ^235^ U ) required to maintain the chain reaction and increases the efficiency of the reactor [44]. In spallation neutron sources, research reactors, and reactors for isotope production, they serve to produce maximum neutron fluxes in certain zones, where a neutron guide can be installed to extract neutrons or a sample can be placed for irradiation. Therefore, neutron reflectors play a key role as they increase the performance of neutron sources and transport systems in an economical and efficient manner.

The reflection of neutrons, like, for instance, the reflection of light, can be either specular (the angle of incidence is equal to the angle of reflection; a clear image is formed [45]) or diffuse (the direction of the reflected radiation is not directly related to the angle of incidence and the image is not formed).

The probability of neutron specular reflection is close to unity if the energy corresponding to the neutron velocity component perpendicular to the surface is lower than the critical energy of the substance; it rapidly decreases with an increase in the perpendicular component of velocity above the critical value. Diffuse reflection might occur due to the multiple scattering of the neutron inside the medium. The reflection probability in this case also depends on the angle of incidence, since even with isotropic scattering in the medium, the probability of reflection at grazing angles of incidence should be greater than at normal incidence because the neutron makes its first collision at a shallower depth. The reflection probability in this case is usually called the albedo [46].

In some cases, specular reflection is of key importance. For example, it is essential in neutron guides [47], which are used to extract neutrons from the reactor over considerable distances (sometimes reaching hundreds of meters) with minimal losses in the phase space. Neutrons are repeatedly reflected from the neutron guide walls at small angles, and any non-specular reflection leads to losses. In many other cases, the specularity of neutron reflection is not important, and only the reflectivity of the substances is important, like in the case of reflectors in nuclear reactors [48,49].

The reflection of neutrons can be elastic (conserving the neutron energy in the laboratory reference frame) or inelastic. Thus, neutron mirrors provide elastic reflection, while the inelastic scattering changes the neutron energy. The latter is of fundamental importance, for example, for the operation of nuclear reactors. Since the fission cross-section decreases in inverse proportion to the neutron velocity, below a certain characteristic velocity, cooling the neutrons increases the reactor efficiency. Thus, reflectors often play the role of moderators. The cooling of neutrons, of course, is possible only under the condition that the moderator temperature is lower than the neutron temperature. For cooler neutrons, an effective reflector must provide elastic reflection (since inelastic reflection would mean the heating of the neutrons) or cooled down to/below the neutron temperature. Different ranges of neutron velocities are discussed below.

The properties of neutron reflectors are directly related to the wave properties of the neutron. If the neutron wavelength is much smaller than the interatomic distance in the medium, which is typically ∼0.2 nm, then the motion of the neutron in this medium is determined by neutron scattering on the nuclei of individual atoms. In this case, neighboring nuclei do not affect the probability and character of scattering. Such a pattern is observed in the energy range of epithermal and fast neutrons ( $E_{n}≳1$ eV).

As a result of multiple scattering in the medium, neutrons, if not captured by nuclei, can return to the half-space from which they came, in other words, be reflected from the medium. Such a reflection will obviously be diffuse. Its probability depends on the ratio of the transport cross-section and the loss cross-section. The transport cross-section increases with an increase in the scattering cross-section and as the angular distribution of neutrons after scattering approaches isotropic.

If the neutron wavelength is close to the interatomic distances in the medium or greater than them, then the motion of the neutron is the result of the interference of neutron waves scattered on neighboring nuclei. In the energy range of thermal neutrons (∼ $10^{-3}$ eV $<E_{n}<1$ eV), the dominant process in ordered media is coherent Bragg–Wulf scattering [50,51], which is a reflection from a large number of crystalline planes at once. In this case, the probability of neutron scattering in the substance (and, accordingly, the albedo) increases. The boundaries of the interval are not precisely defined and simply rounded to whole numbers. In particular, for the lower boundary it is due to the fact that there are substances with different inter-planar distances, and the scattering intensity gradually decreases with increases in the wavelength. As reflectors of thermal neutrons, substances consisting of atoms with a small capture cross-section, such as graphite and beryllium, as well as heavy and light water, are most often used. The maximum albedo for thermal neutrons is observed for heavy water and reaches ∼98%. However, to achieve such high albedo values, large thicknesses of heavy water are required (∼1 m). In addition, the neutron changes its energy during such a reflection. Only the Maxwellian spectrum of neutrons in equilibrium with the reflector will retain its temperature upon reflection.

If the neutron energy decreases below ∼ $10^{-3}$ eV (CN energy range), the neutron wavelength increases so much that it ceases to distinguish the inhomogeneity of the medium consisting of individual atoms, and interference on them leads to the appearance of the so-called optical potential [52]. As a result, the medium becomes practically transparent for the neutron (it only slightly changes its energy by the value of the optical potential). This phenomenon is widely used to design CN filters [53,54].

The value of the optical neutron–nuclei potential (or the critical energy or Fermi potential) of a substance varies for different substances from about −100 neV to about +300 neV (we can also talk about the critical velocity of a substance, i.e., the velocity of a neutron, which has a kinetic energy equal to the critical energy of the substance) [55,56]. If a neutron has an energy below the critical energy of the substance (the energy range of ultracold neutrons (UCNs)), then it cannot penetrate into the substance from a vacuum at any angle of incidence and is reflected from it with a probability close to 100% while not changing its energy. This reflection process is very similar to the total internal reflection of light. The theoretical probability of UCN losses due to the reflection can be small and reach ∼ $10^{-9}$ ; however, in experiments, minimal losses were observed only at the level of ∼ $10^{-5}$ – $10^{-6}$ . Still, such low losses make it possible to store UCNs in closed traps for a long time (almost equal to the neutron lifetime) as an ordinary gas [57,58,59,60].

If a neutron with an energy above the critical energy of the substance falls on the surface at a certain angle, and if the normal component of the neutron velocity to the surface is smaller than the critical velocity of the substance, then the neutron will also be reflected specularly from the surface like an UCN. This is how mirror neutron guides work. In this case, the greater the critical energy of the material of the neutron guide, the greater the angles of neutron reflection and the greater the density of the neutron flux will be in the neutron guide. The maximum critical velocity, ∼7–8 m/s, is provided by beryllium, diamond, and ^58^ Ni coatings.

One can significantly increase the effective critical velocity of the neutron guide coating by using a multilayer structure, which has alternating layers of substances with positive and negative critical energies. The thicknesses of the layers vary with the depth of the layer and are made such that a neutron with any energy, as it penetrates deep into this structure, could “find” several successive layers for which, at its energy and angle of incidence, the Bragg–Wulf condition was satisfied, and it has a quite high probability of being reflected from them. Such a structure is called a supermirror [61].

The production of supermirrors is very laborious, since it requires the deposition of hundreds or even thousands of strictly calculated layers, the number of which grows very rapidly as the effective critical velocity of the surface increases. In addition, the probability and degree of specular reflection of a neutron decreases significantly with an increase in the number of layers. Most often, supermirrors are used that increase the critical velocity of the surface of natural nickel by a factor of 2 (the so-called m=2 supermirrors, where *m* is the ratio of the effective critical angle/velocity of the supermirror to the critical angle/velocity of natural nickel), up to ∼15 m/s. The record belongs to a supermirror with a critical velocity of ∼55 m/s (m=8) [62]; however, the probability of neutron reflection with the maximum velocity from this range becomes lower than ∼ 40% . In addition to the complexity of manufacturing, the reflection from supermirrors is not purely specular. This deviation from specularity is small in the range of thermal neutrons but becomes significant in the VCN range due to a closer match between the characteristic sizes of inhomogeneities in supermirrors and the VCN wavelengths.

**Figure 1:**
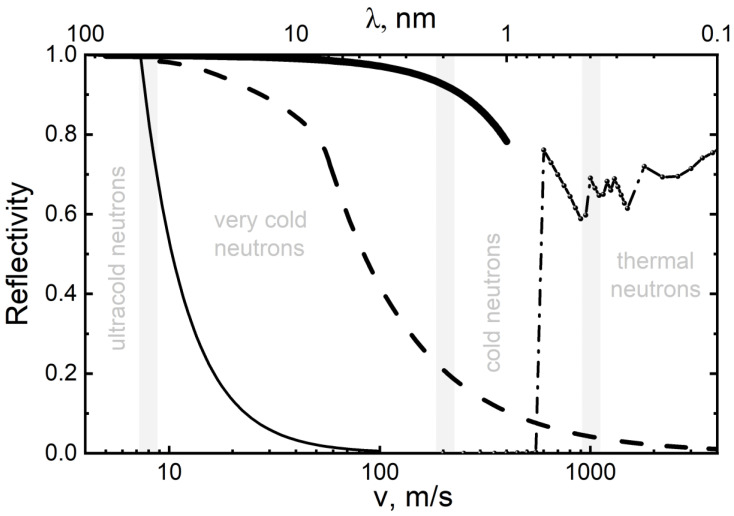
The reflection probability for an isotropic neutron flux as a function of neutron velocity (bottom scale) and wavelength (top scale) for various carbon reflectors: (1) diamond-like coating (DLC) (thin solid line), (2) the best supermirror [62] (dashed line), (3) hydrogen-free DND powder of infinite thickness (thick solid line), (4) calculation using the Monte Carlo N-Particle^®^ (MCNP) standard program for reactor graphite reflector [44] with infinite thickness at ambient temperature (dashed–dotted line). The reflection is elastic except for the last curve where only the fraction with no increase in energy is taken into account. Velocity (wavelength) ranges of ultracold, very cold, cold, and thermal neutrons are separated by broad gray vertical lines.

The performance of existing neutron reflectors is illustrated in Figure 1, which shows the probability of reflection of neutrons of various energies from the three types of carbon reflectors indicated above in the case of an isotropic incidence of neutrons on the surface. Since the diffuse reflection of thermal neutrons is not strictly elastic (due to the recoil of nuclei and scattering by phonons), the figure shows the probability of reflection for it without increasing the neutron energy; the total reflectivity is higher. The energy ranges mentioned above are indicated in this figure in terms of velocities.

In this section, several groups of neutrons were mentioned that differ in their characteristic energies/velocities/wavelengths: thermal neutrons, CNs, VCNs, and UCNs. The boundaries of these groups are not clearly defined, even if there is a physical reason for their selection.

So, the boundary between thermal neutrons and CNs is determined by the presence/absence of Bragg scattering at large angles [50]. This scattering disappears when the neutron wavelength becomes larger than twice the maximum distance between crystal planes. Since different crystals have different structures and values of their parameters, the boundary varies from crystal to crystal. It is generally accepted to consider the wavelength of ∼0.4 nm as the upper boundary of CNs, where the Bragg scattering disappears for a beryllium poly-crystal. Beryllium is often used to isolate CNs via transmission because it has a small capture cross-section; the cooling of beryllium is intended to suppress scattering on phonons by freezing them out, thereby reducing the scattering cross-section and increasing the transmission.

The situation is similar with UCNs. The UCN upper boundary corresponds to the possibility of storing neutrons, i.e., when the kinetic energy of the neutron is smaller than the value of the optical potential. As in the previous case, the magnitude of the potential differs considerably for different substances. In the literature, different values are taken as the upper boundary of UCNs, corresponding to the optical potential of beryllium ( 6.89 m/s, 248 neV, 57.5 nm), natural nickel ( 6.84 m/s, 245 neV, 57.9 nm), and isotope ^58^ Ni ( 8.14 m/s, 346 neV, 48.6 nm).

The situation is even more complicated with the definition of the upper boundary of thermal neutrons and the separation of CN and VCN ranges. In the first case, the conventional value of ∼ 0.4 eV (corresponding to the resonance in the ^113^ Cd capture cross-section) is often taken as the boundary. In the second case, there was no physical reason for the distinction. VCNs are often understood as either neutrons that are already practically absent in CN beams (neutrons with wavelengths over ∼ 2.0 nm) or neutrons that are still present in the only operating VCN beam [63] (wavelengths over ∼ 2.5 nm). We propose to define VCNs as neutrons that are effectively reflected from ND powders that also correspond to ∼2.0–2.5 nm.

It is useful for the following to provide the boundaries of these groups (taking into account all the reservations made above) in terms of neutron wavelengths and characteristic velocities:

– Thermal neutrons ∼ 0.05 nm (8000 m/s) <λ< 0.4 nm (1000 m/s), v∼2200 m/s;
– CNs ∼ 0.4 nm (1000 m/s) <λ< 2 nm (200 m/s), v∼500 m/s;
– VCNs ∼ 2 nm (200 m/s) <λ< 50 nm (8 m/s), v∼50 m/s;
– UCNs ∼ 50 nm (8 m/s) <λ , v∼5 m/s.

As can be seen from Figure 1, there is a large gap in the reflectivity of existing reflectors in the range of VCNs and CNs. A series of papers [20,64,65,66,67,68,69,70,71,72,73,74,75,76,77,78,79,80,81,82,83,84] explores the possibility of designing effective VCN reflectors in this so-called reflectivity gap in order to increase VCN fluxes extracted from neutron sources and, possibly, to provide the storage of VCNs in closed traps for research. The solution of this problem opens up prospects for the wide use of VCNs in neutron physics.

In addition, it turned out that the proposed VCN reflectors can be used to reflect CNs at small grazing angles. In this case, an effect is observed that has been called “quasispecular reflection (QSR)”— reflection in which the reflected beam has a relatively narrow angular distribution with a maximum in an approximately specular direction. In this case, the reflection angles turn out to be comparable with the angles for supermirrors with large *m* values, and the angular distribution of reflected neutrons depends on the neutron wavelength/velocity and reflector parameters. The reflector material itself is radiation-resistant. Thus, these reflectors can also be useful for designing systems for the extraction of CNs from sources.

Research on NDs as neutron reflectors is carried out mainly experimentally. The theoretical study, as a rule, was limited to the consideration of the interaction of VCNs and UCNs with a single nanoparticle surrounded with a large structureless medium with some potential; then, the applicability range of the approach used is found. A similar consideration of the interaction of neutrons with nano-objects is used in the theory of small-angle neutron scattering (SANS) [85], where the task is formulated in a certain sense as the opposite: to obtain information about particles from the scattered signal and not to search for ways to change the parameters of this signal. For thermal neutrons and CNs, two physical phenomena, i.e., scattering on the particle structure and scattering on the particle as a whole, are often considered independently. The problem of neutron scattering has not yet been solved in a general form due to its complexity. Attempts are being made to design adequate models for calculating the transport of neutrons in ND powders, which could be used to simulate devices based on ND reflectors [73]. In some cases, theoretical results from other areas of physics (for example, optics) can be used, which can be generalized when the characteristic behavior of waves of different natures is considered.

## 3. Industrial NDs as VCN and CN Reflectors

The idea of using nanoparticles to reflect long-wavelength neutrons seems extremely simple. As noted in the previous section, due to the large de Broglie wavelength of VCNs, the result of the interaction of such neutrons with a homogeneous medium is their forward scattering. The interaction can be changed by making the medium inhomogeneous on a scale of the order of a wavelength. For VCNs, this scale is on the order of nanometers, and it would seem that the study of the possibility of using nanopowders to reflect VCNs should have been completed long ago. In addition, neutrons (thermal and CN) have long been widely used to study nanoparticles via the SANS method.

The reason for this delay seems to be that researchers involved in the study of CN and thermal neutron scattering on nanoparticles were not interested in the field of research with VCNs, and physicists working with UCNs did not use nanoparticles. To be fair, it should be noted that the latter made attempts to expand the range of velocities of stored neutrons by using the reflection of above-critical neutrons from an inhomogeneous medium. Back in the 1970s, A. Steyerl noticed the possibility of storing VCNs in a trap made of pyrolytic graphite [86]. In work [87], it was proposed to “replace” individual atoms with nanoparticles, thereby greatly increasing the efficiency of neutron scattering by increasing both the coherent scattering cross-section and the effective size of the scatterer. The Fermi theory of nuclear reactor moderators/reflectors was used to describe the interaction of VCNs and CNs with such nanoparticles. V. Morozov’s group showed reflection coefficients close to unity for various media inhomogeneous on this scale using neutrons with velocities up to ∼10 m/s [88] and even made a first model analysis of the use of NDs for reflecting VCNs based on the diffusion approximation [89], but they did not begin to study this problem experimentally. The reflection of the entire range of VCNs and CNs from ND reflectors was considered theoretically in ref. [90].

The impetus for experimentally studying the possibility of designing VCN reflectors based on NDs was ref. [91]. Here, UCNs were used as a mechanism of “small heating” or UCN reflection from trap walls with an increase in neutron energy by ∼ $10^{-7}$ eV [87].

As already noted, when describing the interaction of VCNs with matter, Bragg scattering can be ignored. Let us consider, for simplicity, the scattering of a neutron by one homogeneous spherical particle of radius *R*, consisting of ∼ $10^{3}$ – $10^{5}$ atoms. The incident neutron interacts only with the mean potential of the particle, which can be written as follows:

(1) $$V\left(\vec{r}\right)=\{\begin{array}{cc} V=V_{0}-iV_{1}, & if\;r<R\\ 0 & if\;r>R\;. \end{array}$$

*V* is the (complex) optical Fermi potential [52] averaged over the volume of the particle for each nucleus:

(2) $$V_{0}-iV_{1}=\sum_{j}\rho^{\left(j\right)}\int V^{\left(j\right)}\left(\vec{r}\right)d\vec{r},$$

where the summation is carried out over different types of nuclei in the particle, in each term of the sum $\rho^{\left(j\right)}$ is the number of nuclei of type (j) per unit volume, and $V^{\left(j\right)}\left(\vec{r}\right)=V_{0}^{\left(j\right)}-iV_{1}^{\left(j\right)}$ is the interaction potential between a neutron and a nucleus of type (j) . The real part of the potential can be obtained from the coherent scattering length $b^{\left(j\right)}$ :

(3) $$V_{0}^{\left(j\right)}=\frac{2\pi h^{2}}{m}\int b^{\left(j\right)}d\vec{r},$$

where *m* is the neutron mass. The imaginary part of the potential, which describes the neutron capture, can be calculated from the absorption cross-section using the optical theorem [92], taking into account the fact that this interaction is not strong and thus can be considered within the first Born approximation [93]:

(4) $$\frac{2m}{\hbar^{2}}\int V_{1}^{\left(j\right)}d\vec{r}=\sigma_{a}^{\left(j\right)}\left(k\right)k,$$

where $\sigma_{a}^{\left(j\right)}$ is the capture cross-section for nuclei of type (j) . Experimental data for the capture cross-section are given for thermal neutrons, i.e., for a neutron velocity of 2200 m/s. Thus, the result can be expressed in terms of $\sigma_{a}^{\left(j\right)}\left(k_{0}\right)k_{0}$ , where $k_{0}$ is the absolute value of the thermal neutron wave vector. As a result, the following expressions for the potentials are obtained:

(5) $$V_{0}=2\pi \frac{\hbar^{2}}{m}\sum_{j}\rho^{\left(j\right)}b^{\left(j\right)},$$

(6) $$V_{1}=\frac{\hbar^{2}}{m}\sum_{j}\rho^{\left(j\right)}\sigma_{a}^{\left(j\right)}\left(k_{0}\right)k_{0}.$$

Using the first Born approximation, one can easily obtain expressions for the scattering amplitude f(θ) in the center of mass reference frame. If $\vec{k}$ and $\vec{k^{\prime }}$ are the neutron wave vectors before and after scattering, then

(7) $$f\left(\theta \right)=-\frac{1}{4\pi }\frac{2m}{\hbar^{2}}\int e^{-i(\vec{k}-\vec{k^{\prime }})r}V\left(\vec{r}\right)d\vec{r},$$

where θ is the angle between the vectors $\vec{k}$ and $\vec{k^{\prime }}$ . Denoting the transferred momentum as $\vec{q}=\vec{k}-\vec{k^{\prime }}$ and taking into account that with elastic scattering in the center of mass reference frame $k=k^{\prime }$ and q=2ksin(θ/2) , we finally obtain the following:

(8) $$f\left(\theta \right)=-\frac{2m}{\hbar^{2}}VR^{3}\frac{1}{\left(qR\right)}j_{1}\left(qR\right),$$

where $j_{1}\left(x\right)=sin\left(x\right)/x^{2}-cos\left(x\right)/x$ is the first spherical Bessel function.

Knowing the amplitude, one can calculate the scattering cross-sections $\sigma_{s}$ and capture $\sigma_{a}$ on a particle in the center of mass system:

(9) $$\sigma_{s}=\int {\left|f\left(\theta \right)\right|}^{2}d\Omega =2\pi {\left|\frac{2m}{\hbar^{2}}V\right|}^{2}R^{6}\frac{1}{{\left(kR\right)}^{2}}I\left(kR\right),$$

where

(10) $$I\left(kR\right)=\int_{0}^{2kR}\frac{1}{x}j_{1}\left(x\right)dx=\frac{1}{4}\left(1-\frac{1}{{\left(kR\right)}^{2}}+\frac{sin\left(2kR\right)}{{\left(kR\right)}^{3}}-\frac{{sin}^{2}\left(kR\right)}{{\left(kR\right)}^{4}}\right).$$

To obtain an expression for the absorption cross-section, we use the optical theorem: $Im(f\left(\theta =0\right)=k\sigma_{a}/4\pi$ . Since from Formula (8) it follows that $f\left(0\right)=\left(-1/3\right)\left(2m/\hbar^{2}\right)VR^{3}$ ,

(11) $$\sigma_{a}=\frac{4\pi }{3}\frac{2m}{\hbar^{2}}V_{1}R^{4}\frac{1}{kR}.$$

Using Expressions (5) and (6), the potential parameters for nanoparticles from various materials with a small neutron capture cross-section were calculated. These results are presented in Table 1.

Formula (9) shows that the scattering cross-section $\sigma_{S}$ is proportional to the square of the potential $V^{2}$ . The materials listed in the first four rows of Table 1 require low temperatures (below 19 K, 277 K, 55 K, and 195 K, respectively) to exist. From the bottom two lines, it clearly follows that diamonds are a much more promising material than beryllium.

If the goal is the maximum probability, then one prefers particles of a size that provides the maximum transport cross-section $\sigma_{tr}=\sigma_{s}\;\left(1-<cos\left(\theta \right)>\right)$ , which depends both on the value of the scattering cross-section $\sigma_{S}$ and on the mean cosine of the scattering angle <cos(θ)> . It is intuitively clear that this optimum lies in the range of dimensions on the order of the wavelength of the reflected neutrons, i.e., in the nanometer size range. On the one hand, a decrease in size leads to a sharp drop in the scattering cross-section, according to Expression (9) (the cross-section drops as ∼ $R^{4}$ for λ≪R and ∼ $R^{6}$ for λ≫R ). On the other hand, with an increasing particle size, the mean cosine of the scattering angle sharply decreases.

The mass of an ND with a radius of 1 nm is ∼ $10^{5}$ times greater than the mass of the neutron. Thus, the recoil of the ND during neutron scattering can be neglected, and the scattering can be considered absolutely elastic. The velocities of motion of such a free ND at room temperature are on the order of several m/s , and they rapidly decrease with an increasing ND size, ∼ $R^{3⁄2}$ , and with decreasing degrees of freedom of the ND during motion, i.e., with an increase in the effective mass of the ND. However, since the number of scattering events can be large upon reflection from a medium, the possibility of a change in the velocity of neutrons upon reflection due to the Doppler effect should be given attention.

To estimate the optimal size of the ND, one can use the known solutions for the albedo from a semi-infinite medium obtained by solving the diffusion equation (see, for example, [94]), which, in the limit of small losses in the medium, coincides with the solution obtained via the algebraic calculation method [95]:

(12) $$R_{\infty }=\frac{\sqrt{\sigma_{a}+\sigma_{tr}}-\sqrt{\sigma_{a}}}{\sqrt{\sigma_{a}+\sigma_{tr}}+\sqrt{\sigma_{a}}}.$$

Using (12) and expressions for the corresponding cross-sections in the Born approximation, one can estimate the optimal ND diameter, which corresponds to the maximum albedo. For diamond spheres, the optimal ND diameter $D_{opt}=2R_{opt}$ is related to the neutron wavelength λ and neutron velocity *v* as follows [90]:

(13) $$D_{opt}\;\left[\mathrm{nm}\right]\approx 0.54\lambda \;\left[\mathrm{nm}\right]\approx 215/v\;\left[m/s\right].$$

In particular, for a typical VCN velocity of ∼50 m/s, the optimal ND diameter is ∼ 4.3 nm.

**Figure 2:**
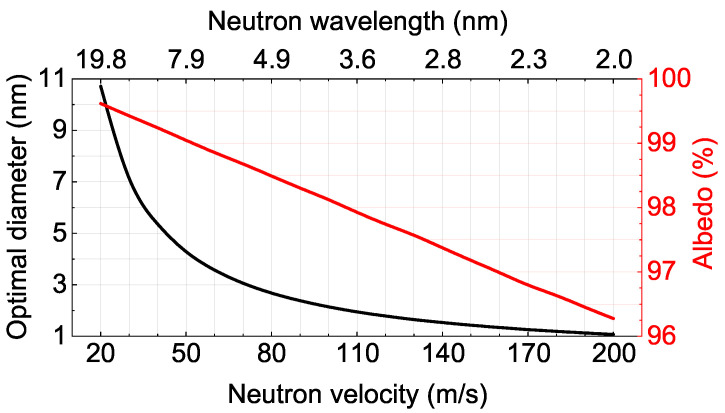
Optimal diameters of diamond nanospheres (black line) as a function of VCN velocity (bottom scale) and wavelength (top scale). The solid red line shows the corresponding calculated VCN albedo for the optimal diameters of diamond nanospheres. Only neutron absorption by carbon is taken into account. The layer of DND is of the infinite thickness.

Figure 2 illustrates the calculated optimal ND sizes and the corresponding maximum albedo values for a semi-infinite medium as a function of neutron velocity in the range from 20 m/s to 200 m/s and a corresponding wavelength range.

Thus, the simple estimates show that NDs are the most promising material for designing a VCN reflector due to the unique combination of their properties: a large amplitude of coherent scattering, a small capture cross-section for carbon atoms, and a high nuclear concentration of these atoms in the diamond phase. A sufficiently thick layer of ND powder with a particle size (diameter) of several nm should give a large albedo for VCNs. At the same time, this powder will be inefficient for thermal neutrons and CNs [90]. Of course, the real situation is much more complicated, since the simplest consideration presented above did not take into account that real NDs can have different shapes, and NDs in a powder can have a certain size distribution and can be combined into agglomerates, etc. As can be seen from Figure 2, the optimal sizes of NDs for reflecting neutrons with velocities of 30–110 m/s lie in the range of 7–2 nm. Luckily, the main amount of NDs produced via detonation synthesis lies in the same range.

**Figure 3:**
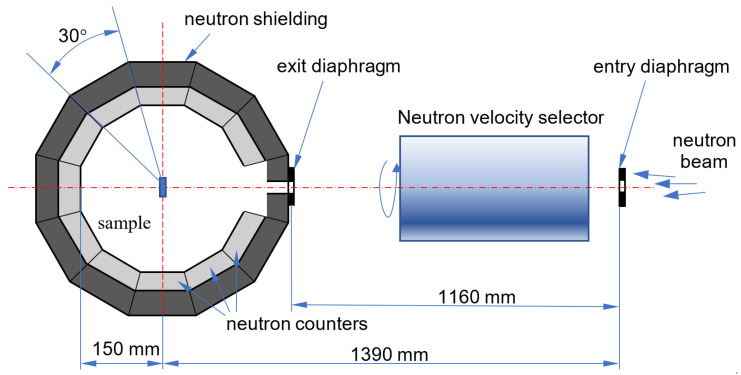
A scheme (top view) of the installation used to measure neutron reflection/transmission from/through the ND layer. Two diaphragms (entry and exit) form the neutron beam in a horizontal plane. A mechanical neutron velocity selector is installed between the two diaphragms; it passes a band in the neutron velocity spectrum with a resolution of ∼ 20% . The sample is placed in the neutron beam after the exit diaphragm so that the neutrons are incident along the normal to the plane of the sample, in the center of the detecting system, which consists of 11 neutron counters that detect the neutrons scattered on the sample. The geometry of the detecting system is such that the flat rectangular input windows of the counters are the side faces of a regular rectangular prism, at the base of which lies a regular dodecagon on a horizontal plane. The neutron beam passes through the center of one of the faces of the prism (free from the counter) perpendicular to this face. The input window of the counter, which forms the opposite face, measures the transmitted neutron beam. Each of the remaining ten detectors overlaps in the horizontal plane the corners with vertices in the center of the sample at ∼30° (∼28° taking into account the thickness of the side walls of the counter) and ∼60° in the vertical plane (the solid angle of ∼ π/6 ). The entire detecting system is surrounded by neutron shielding. To reduce scattering in the air, the entire detecting system (together with the sample placed at its center) was placed in an argon atmosphere.

**Figure 4:**
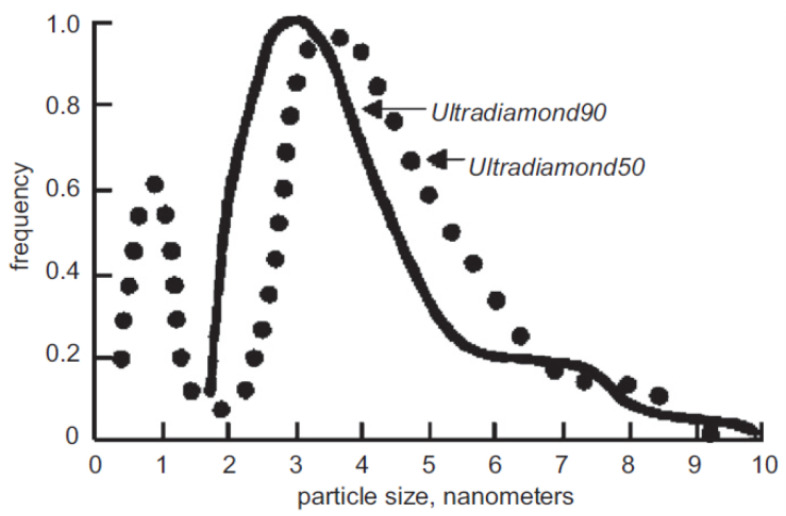
Size distribution of DNDs is provided by the producer (Ultradiamond, USA).

To test this assumption, the transmission/reflection of VCNs through/from the thin DND layer was measured [64] using the scheme shown in Figure 3 (top view). The sample was DND powder (Ultradiamond90, Ultradiamond, USA) with the size distribution shown in Figure 4.

The powder was placed between two thin plane-parallel sapphire plates. Four samples were prepared with thicknesses of 0.2 mm, 0.4 mm, 2.0 mm, and 6.0 mm, respectively. The choice of thickness was determined by the following considerations: the thinner the sample, the lower the effect of multiple scattering. Thinner samples thus make it possible to study the interaction of a neutron with NDs. The thicker the sample, the greater the reflectivity. Thus, thicker samples should make it possible to assess the feasibility of using ND powder as reflectors. The minimum thickness of the powder layer at which the sample is homogeneous (without through holes) was 0.2 mm; the measured density of the samples turned out to be 0.60±0.03 g/cm^3^.

**Figure 5:**
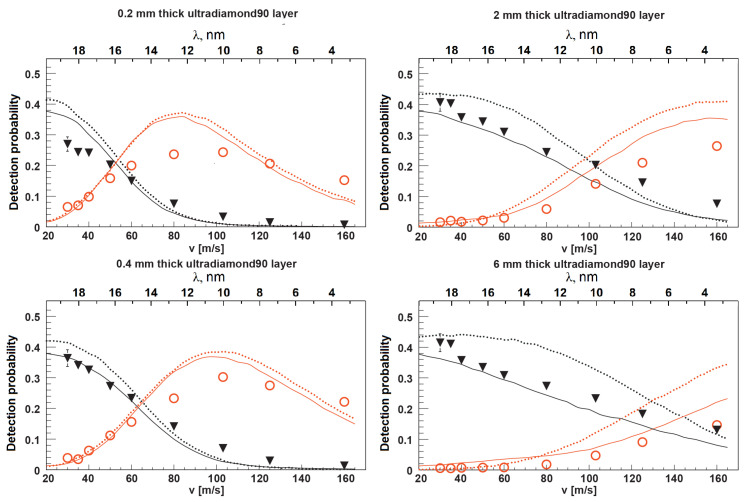
The probability of detecting VCNs scattered on an Ultradiamond90 sample (normalized to the incident neutron flux) into the forward hemisphere (the sum of events in the detectors located at the angles of 30°, 60°, 300°, and 330°) and the rear hemisphere (the sum of events in the detectors located at the angles of 120°, 150°, 210°, and 240°) as a function of VCN velocity (bottom scale) and wavelength (top scale), for various powder thicknesses. Triangles correspond to the measured VCN backscattering probability. Circles stand for the VCN forward scattering probability. The dotted lines correspond to the model of independent nanoparticles at rest without taking into account the losses due to hydrogen impurities. Solid lines indicate the model taking into account the “heating” of VCNs on hydrogen impurities. Dotted lines correspond to the model ignoring the “heating” of VCNs on hydrogen impurities. The experimental scheme is shown in Figure 3. A similar figure is given in ref. [64].

The results are shown in Figure 5. Expressions (9) and (11) were used to calculate the scattering and capture cross-sections. The supposed chemical composition of the NDs included carbon (up to 88% of the total mass), hydrogen ( 1.0% ), nitrogen ( 2.5% ), and oxygen (up to 10% ) [96]. Since the amount of hydrogen (both chemically bound and sorbed in the form of water) was not known exactly, this amount was considered as a free parameter of the model. The amount of hydrogen was evaluated approximately as expected. The main uncertainty was associated with inaccurate knowledge of the cross-section for neutron scattering on hydrogen, which depends on the chemical bonding and was later studied experimentally [97]. In the simulation, it was assumed that hydrogen is uniformly distributed in the sample, and the dependence of the cross-section on the velocity *v* for (isotropic) inelastic neutron scattering follows the law 1/v . It was also assumed that neutrons leave the sample immediately after an inelastic scattering event. The difference between the experimental results and the model calculation can be considered as an indicator of the degree of reliability of the model.

Using the time-of-flight method (a chopper was placed between the selector and the exit diaphragm), it was verified that the neutron reflection was indeed elastic. It follows from the results of this measurement that the total average change in velocity as a result of scattering (single or multiple) on the sample does not exceed ±1 m/s.

A good qualitative agreement between the measurement results and the predictions of the model of independent nanoparticles at rest was obtained over a wide range of VCN velocities and sample thicknesses. This agreement allows us to conclude that the total probability of VCN reflection at the lowest velocities is close to 100% (taking into account that some of the neutrons are scattered in directions not covered by the counters).

Using the described model, the albedo from flat powder layers was calculated for an isotropic neutron flux as a function of their velocity. The results of these calculations are shown in Figure 1 (the effect of hydrogen was not taken into account). As can be seen from Figure 1, the maximum energy of VCNs reflected by the powder and the probability of reflection are much higher than the corresponding values for the best available supermirrors [62]. These estimates showed that the reflector made of NDs can be used to significantly increase the flux of VCNs and, possibly, store VCNs in closed traps.

After the observation of the effective reflection of neutrons from the DND powder, we wanted to precisely measure the albedo from the powder. In order to collect all the reflected neutrons in the experiment described above, you need to build a detector with a solid angle ∼ 2π and evaluate the absolute efficiency of collecting scattered neutrons, taking into account the “dead zones” of the detector. Such zones, i.e., the zones where the detector does not count neutrons, are inevitable, at least to allow the incident beam to pass through the detector. Reaching the required accuracy for the albedo in such an experiment seems to be a difficult experimental problem when its value differs from unity by several percent only. In addition, the albedo and angular distribution of the reflected neutrons can depend significantly on the angle of incidence of the neutron beam. Thus, the experiment would have to be repeated for different angles of incidence of the neutron beam.

**Figure 6:**
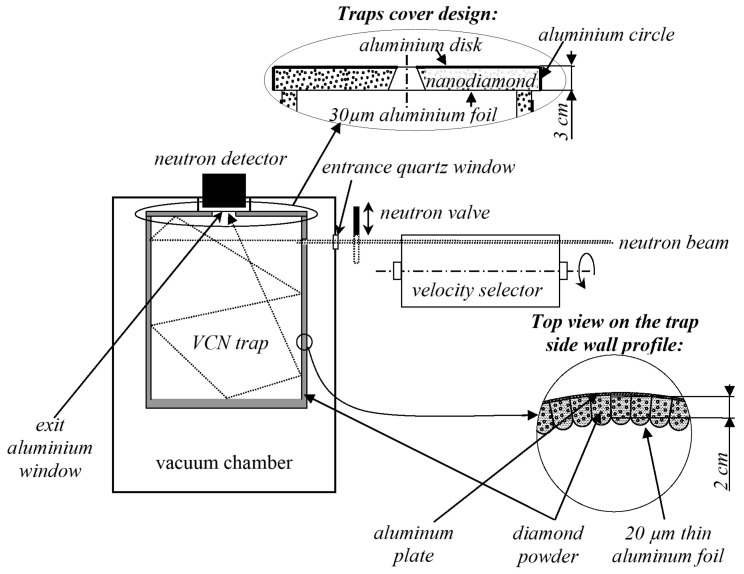
A scheme of the VCN storage experiment [65]. A collimated neutron beam of ∼1 cm in diameter enters the cylindrical cavity of the VCN trap with a diameter of ∼44 cm and a height of ∼47 cm, through a small hole (∼ 2×2 $\;cm^{2}$ ) in the side wall. VCNs, being repeatedly reflected from the VCN trap walls, can enter the exit hole with a diameter of ∼6 cm in the trap ceiling and be counted in a neutron detector located behind the hole. The incoming neutron beam can be closed or opened with a neutron valve. The velocity of VCNs in the beam is set with a velocity selector located in front of the neutron valve. The VCN trap is placed in a vacuum chamber with an input quartz window and an output thin aluminum window. If the VCN beam is closed, the count rate in the detector decreases exponentially, following the VCN density in the trap. Thus, one could measure the storage time of VCNs as a function of their velocity and wavelength. The experiment consists of measuring the decay time constant of the count rate in the detector after the neutron beam is closed at the entrance to the trap, as a function of the VCN velocity/wavelength. A similar figure is given in ref. [65].

To avoid these problems, we used a method for measuring the neutron storage time in a closed volume, borrowed from UCN physics (see, for example, [59,60]). This method allows us to evaluate the loss factor (the difference between the loss probability and unity) at an accuracy of ∼ $10^{-5}$ . Taking into account that the velocity of VCNs is 10–100 times larger than that of UCNs and the reflection coefficient would not be so high, the expected storage times would be significantly shorter than those in experiments with UCNs; this difference had to be taken into account when planning the experiment. Therefore, we had to increase the trap size, and hence the amount of DND powder. For the implementation of this experiment, a commercially available DND powder (described in particular in ref. [76]) was purchased. The measurements were carried out at the PF2 VCN beam at the ILL reactor (Grenoble, France). A scheme of the installation is shown in Figure 6.

**Figure 7:**
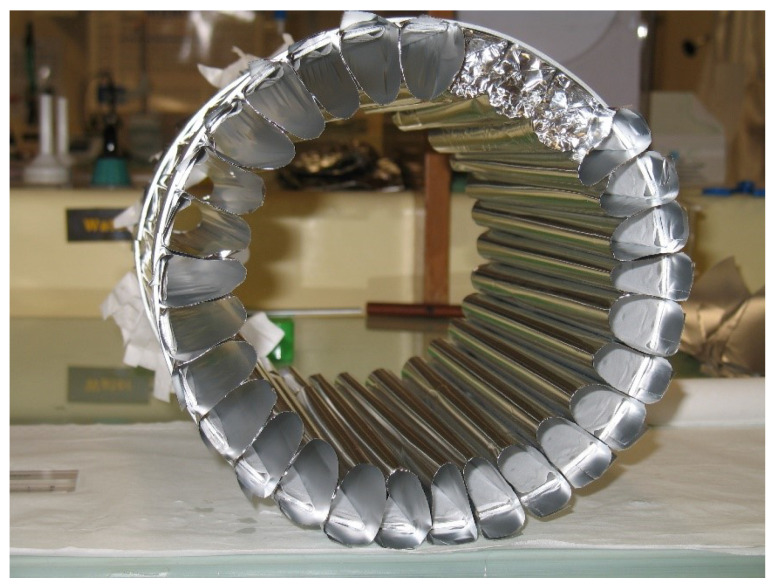
A prototype of the side wall of the trap for storage of VCNs used in the experiment [65]. A stage of manufacturing thin-walled tubes.

The main element of the installation is the neutron trap with walls made of DND powder. Several unsuccessful attempts had been made to fabricate the vertical walls of the trap in such a way that the powder surface is open and the powder structure is strong. For this purpose, the powder was compacted, wet with various solutions followed by their evaporation, etc., which should have led to the “sticking” of the powder. However, cracks invariably appeared after drying. The only way to solve this problem was to put the powder into some kind of thin-wall cover made of a weakly absorbing material in order to limit VCN losses. The details are given in [65]. First, a small prototype was made. Figure 7 shows a stage of assembly of the side wall prototype illustrating the technology of manufacturing tubes.

During the visual control of the powder during pumping it out, it was found that no movement of its parts is observed if the cover is wide. However, if the powder is placed into in a long and thin tube, then a sudden “blowing out” of the powder occurs at a pressure of ∼2–3 mbar; it cannot be significantly affected by reducing the pumping rate. This phenomenon is probably associated with the desorption of water from the DND surfaces. This problem is solved if the surface of the powder is tightly pressed by some kind of gas-permeable filter. The lid of the trap was made in the form of an aluminum circle with a flare, on which thin aluminum foil was stretched; powder was sprinkled on the foil. The mean thickness of the powder was ∼3 cm. In the center of the circle, there was a hole for neutrons to flow out onto the detector. The bottom of the trap was a layer of powder ∼3 cm thick.

The measurements consisted of evaluating the time constant of the decrease in the density of the VCNs in the trap after it ceases to be filled. The measurement cycle consisted of filling the trap with VCNs (with the shutter open) to the equilibrium density and measuring the time dependence of the detector count rate after closing the input shutter. The detector count rate is proportional to the VCN density in the trap. Having evaluated the time constant of VCN storage in the trap, one can calculate the probability of VCN loss and, accordingly, the probability of reflection from the walls.

**Figure 8:**
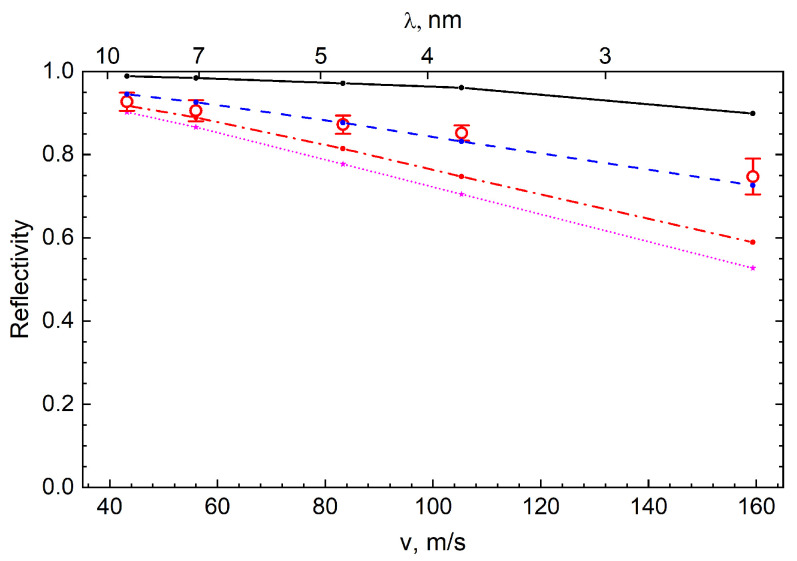
Probability of VCN reflection from a layer of DNDs at ambient temperature as a function of their velocity and wavelength. Open circles correspond to the measurements at room temperature [65]. Thin lines stand for the Monte Carlo calculations taking into account inelastic scattering of neutrons on hydrogen, with different values of inelastic scattering cross-section reduced to the neutron velocity of 2200 m/s: 4b for pink dashed line, 3b for red dashed line, 1.3b for dotted blue line, and 0b for black line. A similar figure is given in ref. [65].

The probability of VCN reflection from the trap wall in this case is equal to the following:

(14) $$P\left(V\right)=1-\frac{\Delta x}{\tau_{st}^{VCN}\left(v\right)v}\left(1-\epsilon \right),$$

where Δx is the average neutron path in the trap between the walls, *v* is the neutron velocity, $\tau_{st}^{VCN}\left(v\right)$ is the measured storage time, and ϵ is a geometric coefficient equal to the ratio of the probability of loss of VCNs in the input and output windows to the total probability of VCN losses in the trap; its value is $3.5\times 10^{-3}$ . The uncertainties in this estimate are given by the uncertainty of the storage time, the mean range Δx , and the neutron velocity. This probability characterizes the reflectivity of the trap walls. It includes losses in the aluminum foil and the effect of the geometry of the trap walls. This estimate assumes an isotropic angular distribution of the VCNs in the trap, which is not entirely true for faster neutrons. For a better assessment, calculations were performed using the Monte Carlo method. The resulting reflectivity is shown in Figure 8 in terms of the albedo as a function of the neutron velocity and wavelength.

In ref. [97], the amount of hydrogen in the powder was measured using the instantaneous γ -quanta from the reaction n(p,d)γ . The results of this measurement showed that for a powder heated in a vacuum or simply evacuated, the ratio of carbon to hydrogen is about 15 to 1 ( $C_{15}H$ ), while for a powder in air this ratio is about $C_{8}H$ .

Figure 8 shows the results of a Monte Carlo simulation (using the mentioned model) of the albedo from the powder at a fixed amount of hydrogen $C_{15}H$ (∼ 0.5% of the total mass). The model assumed that the particle size distribution is the same as for Ultradiamond90 (see Figure 4), the inelastic scattering cross-section obeys the 1/v rule, and the value of the cross-section $\sigma_{ie}^{H}$ for a neutron velocity of 2200 m/s is considered as a free parameter. The calculation reproduces well the dependence of the reflection coefficient on the velocity; the free parameter for the best description of the experimental data is $\sigma_{ie}^{H}=1.3\;b$ .

**Figure 9:**
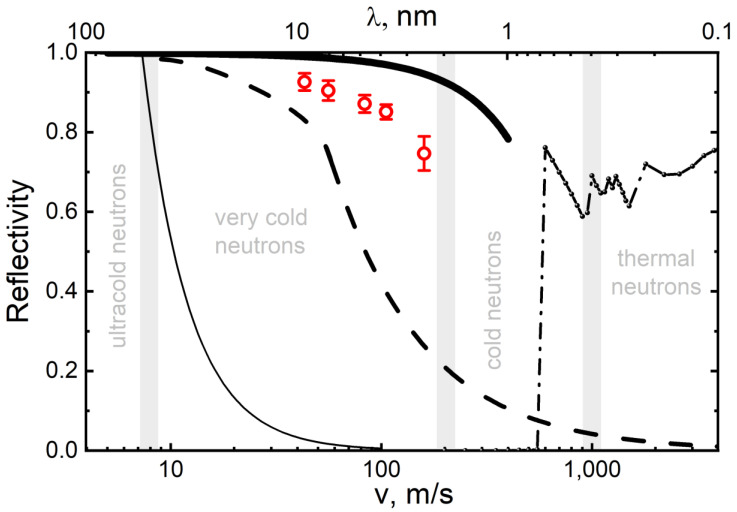
The reflection probability for an isotropic neutron flux as a function of neutron velocity (bottom scale) and wavelength (top scale) for various carbon reflectors: (1) diamond-like coating (DLC) (thin solid line), (2) the best supermirror [62] (dashed line), (3) hydrogen-free DND powder of infinite thickness (thick solid line), (4) DND powder 3 cm thick at ambient temperature (points with error bars) with significant hydrogen contamination, (5) calculation using the Monte Carlo N-Particle^®^ (MCNP) standard program for reactor graphite reflector [44] with infinite thickness at ambient temperature (dashed-dotted line). The reflection is elastic except for the last curve where only the fraction with no increase in energy is taken into account. Velocity (wavelength) ranges of ultracold, very cold, cold, and thermal neutrons are separated by broad gray vertical lines.

The obtained albedo values can be compared with the values for other reflectors, which were shown in Figure 1. The result of this comparison is shown in Figure 9.

As can be seen from Figure 9, the maximum velocity of the reflected VCNs and the measured reflectivity are much higher than those for the supermirrors. Thus, the DND reflector largely bridges the energy gap between the efficient reactor reflectors of thermal neutrons and the UCN optical potential. However, even the ideal DND reflector (thick dashed line in Figure 9) would have to be very thick, for instance, for CNs with a wavelength of 0.89 nm, which is useful for helium UCN sources [98]. Can the range be extended further?

As the neutron velocity increases, the albedo decreases. However, it depends on the angle of incidence of the beam on the surface and, as a rule, it increases with decreases in the angle. Then, the probability of reflection at small grazing angles can also be significant for faster neutrons. Let us consider analogous processes.

The scattering of waves and particles in disordered media is an important research topic in many fields of science [99]. When the characteristic size of the inhomogeneities of the scatterer *d* is much greater than the radiation wavelength λ (this condition corresponds to the case of CN propagation in the DND powder), the wave is scattered on individual inhomogeneities at small angles of the order of $\theta_{eff}∼\lambda /d$ . Thus, the average scattering angle is approximately equal to the ratio of the radiation wavelength to the size of the scatterer. $\theta_{eff}$ is often referred to as the effective SANS angle; the exact form of the angular dependence of scattering depends on the specifics of the scattering process. Such scattering, in particular the scattering of neutrons by inhomogeneities of the neutron nuclear optical potential of a substance, is a powerful method [85,100,101,102] for studying the dimensions, geometric shape, shape of the optical potential, and relative position of scattering centers in the substance. Multiple scattering of CNs by nanoparticles can also be used as a tool [87,90] for particle physics [103,104].

Usually, in a classical SANS experiment, the scattering pattern is recorded after passing neutrons through the sample. However, waves incident on the boundary of a disordered medium at small grazing angles can also be reflected due to multiple small-angle scattering. Examples are the reflection of electromagnetic waves from atmospheric inhomogeneities, aerosols, rain, snow, biological substances, composite materials, etc. [105]. Charged particles (protons, electrons) or neutral particles (atoms) are another example of wave reflection from disordered media. All these and other similar processes are subject to general laws.

The most probable reflection angle and the spread of reflection angles are equal to the angle of incidence, and the probability of such reflection under certain conditions is quite high. For such a reflection, the term “quasi-specular” reflection (QSR) is used [66]. This term emphasizes the main feature of this phenomenon, which is that when a radiation beam is reflected from a flat boundary, most of the reflected radiation is concentrated near the polar angle corresponding to the specular direction with respect to the direction of the incident beam. For any type of radiation with a given wavelength and scatterers of a given size, the probability of such reflection depends on the angular properties of scattering and the ratio of the probability of elastic small-angle scattering to the loss probability, which are determined by specific physical processes. In ref. [106], the process of such reflection is considered analytically in a general form.

Based on the analogy with these processes, it was assumed that DND powders with characteristic nanoparticle sizes of 4–5 nm should quasi-specularly reflect CNs at small grazing angles. Geometric dimensions and shapes [107] of DNDs can be important for optimizing the specific properties of such neutron reflectors, which, in turn, depend on specific applications [80,108]. This is work that must be performed on a case-by-case basis.

As applied to neutrons, the efficiency of the reflection of neutrons from a DND powder is a function of the ratio of elastic SANS cross-sections $\sigma_{c.sc.}$ on the optical potential of the particle to the cross-section $\sigma_{loss}$ of all other processes, which lead to losses, since they reduce the radiation flux in QSR directions [106]. For the neutron, this loss cross-section includes nuclear absorption, incoherent, Bragg, and inelastic scattering:

(15) $$\sigma_{loss}=\sigma_{abs}+\sigma_{inc.sc.}+\sigma_{Bragg}+\sigma_{inel.sc.}.$$

Here, it is assumed that in inelastic, Bragg, and incoherent scattering, the neutron changes direction through a large angle. The interference of neutron scattering on the optical potential and on the crystal lattice of diamonds is not considered. The last simplification is incorrect for neutron wavelengths comparable with the lattice period and should be improved in the future. This interference is negligible at long wavelengths, but is of interest for the characterization of nanopowders at short wavelengths. Thus, the efficiency of QSR is determined by the following relationship:

(16) $$\varepsilon =\frac{\sigma_{c.sc.}}{\sigma_{loss}}.$$

To point out conditions that increase the efficiency of neutron QSR, one needs to know the values of $\sigma_{loss}$ and $\sigma_{c.sc.}$ . The calculation of $\sigma_{c.sc.}$ is easy, and the simplified formalism described above can be used. In the case of VCNs, DNDs should provide a high probability of QSR due to the exceptional combination of properties: a long coherent scattering length for carbon, a high bulk density of diamonds, and low neutron losses. The coherent scattering of neutrons by neighboring DNDs can be neglected, since it is small for the considered case λ≪d . In addition, neutron Mie scattering can be neglected (first analyzed for light scattering by spheres larger than the wavelength of light, in ref. [109]). Mie scattering of neutrons by a single nanoparticle (similar to the phenomenon considered in ref. [110]), in contrast to scattering in the first Born approximation, involves the deflection of some neutrons through very large angles. Such a simplification is justified, since we are interested in SANS, which predominates.

**Figure 10:**
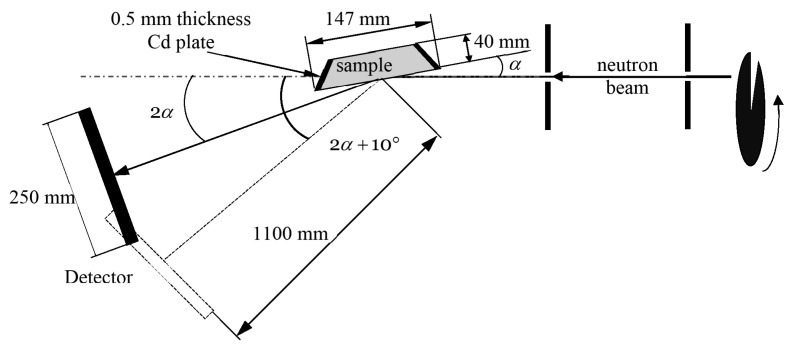
A scheme of the installation (top view) to measure QSR of neutrons. The neutron beam was shaped using two diaphragms. The angular beam divergence in the horizontal plane was ∼ 0.4 mrad; the beam divergence in the vertical plane was ∼2°. The λ -dependence measurements were carried out using the time-of-flight method (using a chopper). Reflected neutrons were recorded in a position-sensitive neutron detector. A similar figure is given in ref. [67].

Schematically, the QSR process is shown in Figure 10. Neutrons arriving at a small grazing angle α can return to the surface after several SANS scatterings. Since the typical number of scattering events is small, the exit angle β is close to α (see Figure 10). In fact, for the case of SANS of CNs on DNDs, the most probable exit angle β is approximately equal to the angle of incidence α with a diffuse halo around this angle [106].

QSR of CNs from DND powders was experimentally studied for the first time in refs. [66,67]. Then, in ref. [80] it was shown how changes in the powder composition and crystallite size affect this reflection.

The scheme of experiments was designed on the basis of the described concept of the effect. The differences in various measurements were only in the studied powders and the method of sample preparation. All measurements mentioned above were carried out at the D17 reflectometer [111] at the ILL. A measurement scheme (top view) is shown in Figure 10.

**Figure 11:**
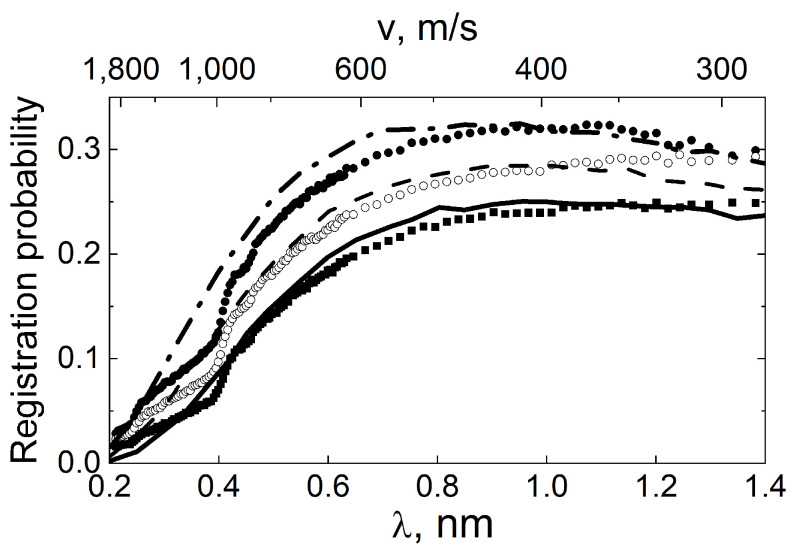
The probability of neutron QSR from a DND sample within the solid angle of the detector as a function of the neutron wavelength (normalized to the incident neutron beam). The grazing angles α are 2°, 3°, and 4° (from top to bottom). Dark and open circles, as well as squares, correspond to the measured data; lines illustrate calculations (for the angles 2°, 3°, and 4° from top to bottom, respectively). A similar figure is given in ref. [66].

In the first measurement, the sample was a DND powder with a density of ∼ 0.4 g/cm^3^ placed in a prismatic container 5 cm high, 15 cm long, and 4 cm deep. The surface of the powder on the side of the neutron beam was covered with a thin aluminum foil. The short vertical sides of the prism were covered with Cd absorbers. The sample temperature could be changed using a special cryostat from 200 °C to the liquid nitrogen temperature. The angular dependence of the QSR probability on the neutron wavelength (in the range of 0.2–2.5 nm) and the grazing angle of the neutron beam to the plane of the sample surface (for the angles of 2°, 3°, and 4°) was studied. The glancing angle α is zero when the neutron beam is parallel to the sample surface; the vertical axis of rotation of the sample table intersects the center of the sample. The spectrum and flux of incident neutrons were measured with the sample moved out of the beam. When measuring the beam reflection, the sample was rotated by an angle of α and the detector by an angle of 2α . For each glancing angle α , the sample was shifted horizontally perpendicular to the beam axis to maximize the flux of neutrons scattered towards the detector. For each glancing angle α , scattered neutrons were recorded at one more position of the detector — the detector was rotated through an angle of 2α+10 °. Thus, two-dimensional distributions of scattered neutrons were measured within a polar angle of 0–24° and a range of azimuth angles from 0° to ±15 °. All measurements were carried out twice: (1) at ambient temperature after the pre-treatment of the sample (long-term annealing and heating at 150 °C under evacuation) and (2) at the liquid nitrogen temperature. Figure 11 shows the probability of QSR into the solid angle of the detector as a function of the neutron wavelength and angle of incidence (see Figure 10). The value of this probability is lower than the actual QSR probability by the fraction of neutrons scattered at large angles that did not fall into the solid angle of the detector.

The fraction of hydrogen atoms in the heated powder remains significant: 1/15. The temperature-dependent inelastic scattering of neutrons by hydrogen is small compared to the temperature-independent elastic cross-section. Any scattering of neutrons by hydrogen (both elastic and inelastic) is isotropic; therefore, it results in the loss of the neutron from the QSR signal. The loss of neutrons on hydrogen was estimated to be 20–40% for various glancing angles. The wavelength range of effective QSR is limited by ∼0.4 nm on the side of short waves due to Bragg scattering in the volume of a DND, which leads to large-angle scattering of short-wavelength neutrons.

Computer simulation of QSR is easy; it is based on Formulas (8) and (11). Modeling is useful for understanding the main features of the phenomenon. The approach used for modeling was tested in previous papers [64]. The geometry shown in Figure 10 was used for modeling. The DND were assumed to be monodisperse. The known parameters were the density of the powder, the fraction of hydrogen, and the scattering cross-sections, which were given above; the unknown parameter was the DND size.

The experimental data were compared with the simulation results within the monodisperse DND model for the neutron incidence angle of 2° and the wavelength λ=1.0 nm. The average diameter of the DND turned out to be 2 nm. This model was extended to other values of neutron incidence angles and wavelengths. The calculated probabilities of hitting the detector are shown in Figure 11 with solid lines. The calculated curves quite well describe the main tendencies in the behavior of the experimental data, with the exception of the range in which the Bragg scattering is significant, which is absent in the calculated model.

**Figure 12:**
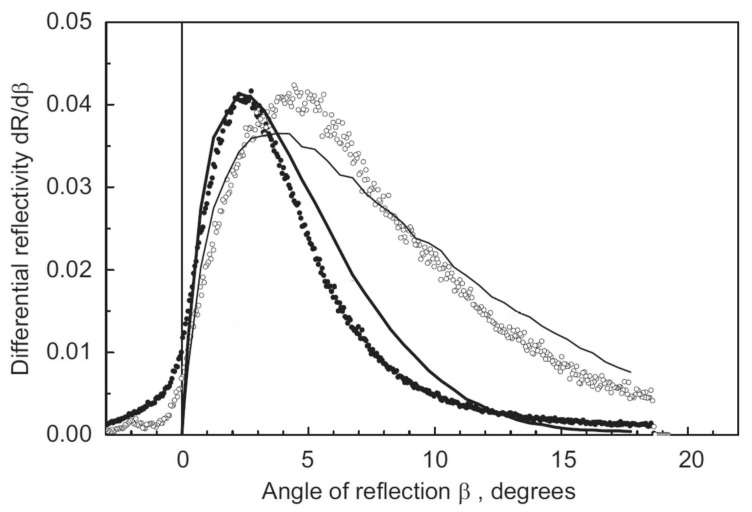
Measured angular distributions of QSR neutrons are shown with dark and open circles, and simulations are indicated by solid lines. The data are averaged over two ranges of incident neutron wavelengths: 0.4–0.5 nm and 0.8–0.9 nm, respectively. The curves are normalized to the incident neutron fluxes in the corresponding wavelength ranges. The neutron beam glancing angle is 2°. A similar figure is given in ref. [66].

Figure 12 shows the measured and calculated angular distributions of QSR neutrons for the neutron beam glancing angle of 2°. Similar wavelength and angle distributions were measured for glancing angles of 3° and 4°. Figure 12 shows that some neutrons reach the detector at scattering angles lower than 0°, which contradicts the geometrical constraint shown in Figure 10. This is probably due to some swelling of the sample during its annealing; the surface was deformed by ∼2 mm. However, this correction is ignored here, since at this stage of this study, the very observation of a new physical phenomenon and the study of its general features were of primary interest and not its precision analysis. The angular distributions of QSR neutrons were calculated using the model described above. Some broadening of the calculated angular distributions in comparison with the measured data can be explained by the simplification of the model (equal sizes of nanoparticles). Nevertheless, there is a fairly good general agreement between the data and the model calculation.

Thus, a new phenomenon of QSR of CNs at small incidence angles from nanodispersed media was experimentally observed for the first time. The features of such reflection are confirmed: the angle of reflection, as well as the half-width of the angular distribution of diffusely reflected neutrons, is approximately equal to the angle of incidence. For an untreated DND powder, the QSR probability within the angular size of the D17 detector for neutrons with a wavelength of 1.0–1.5 nm was ∼ 30% , which corresponds to 40–50% of the total probability (albedo) of QSR. The tangential component of the incident neutron velocity was an order of magnitude larger than the critical velocity for pure diamond.

This phenomenon can be used as a tool for experiments with CNs. For example, it can be used to focus and deliver neutrons and thus complement the capabilities of supermirrors [61] under certain conditions. The results obtained can be used to design and build more efficient neutron facilities and sources.

In conclusion of this section, we note that the first experiments with the reflection of VCNs and CNs from DNDs raised a number of issues that need to be addressed in order to design more effective reflectors. We list the most important of them:

- How can hydrogen atoms (both chemically bound and adsorbed water) be removed from the DND powder? Hydrogen atoms lead to additional neutron losses due to capture and “knock out” from QSR trajectories because of the large incoherent scattering cross-section.
- Is it possible to remove impurities of metals and other elements leading to neutron losses and the potential activation of the DND powder in the radiation fields?
- Will the DND powder be stable in the intense ionizing fields?
- Does the structure of the DND powder affect the efficiency of reflection?
- How will the size of the DND particles in the powder affect the probability and angular distribution of reflection?
- How can the effect of DND powder characteristics on the albedo be studied if a really large amount (kilograms) of powder is needed to accurately measure the albedo?
- Can the density of the DND powder be increased? On the one hand, this increase will allow for a reduction in the thickness of the reflector; on the other hand, with an increase in density, the role of interference effects in scattering by neighboring particles should increase. Obviously, when the density approaches the diamond density, the scattering by particles itself should disappear completely.
- Is it possible to make a non-envelop DND powder reflector through particle sintering/pressing?
- Etc.

Further investigations answered some of these questions, but many questions still remain open and require further study.

## 4. The Problem of Impurities and Its Possible Solutions

One of the advantages of diamond as a neutron reflector material is the extremely small neutron capture cross-section. For thermal neutrons ( v=2200 m/s), it is $\sigma_{a}^{C}∼3.5$ mb. The presence of impurities with a large capture cross-section (for example, the capture by boron atoms is 220,000 times greater than by carbon), even in a small amount, can significantly worsen the reflector efficiency. Therefore, great importance was given to the quantitative study of impurities and the search for ways to reduce them. First of all, the question arose about the possibility of removing hydrogen, as it is the most common impurity with a capture cross-section $\sigma_{a}^{H}∼333$ mb, which is much larger than $\sigma_{a}^{C}$ .

The common methods to study impurities in DND provide relative measurements but experience difficulties in their normalization (IR spectroscopy [112], nuclear magnetic resonance, Inductively Coupled Plasma–Mass Spectrometry [113], Raman scattering, etc). They can be performed with specialized but relatively compact laboratory instruments. The advantage of these methods is that they are sensitive, among other things, to the type of chemical bonds and determine not only the presence of an element but also to which element it is bound and how.

To determine the absolute amount of impurities in the powder, measurements were performed using the neutron activation method, including the method of detecting prompt γ -quanta in a neutron beam [69]. Multi-element instrumental neutron activation analysis (NAA) is a widely used analytical method in various fields of science. Its advantages are a high sensitivity to the presence of impurities, an insensitivity to the type of chemical compound, and the ability to study the sample without a preliminary chemical treatment (for example, ICP-MS assumes the preliminary dissolution of the sample). A disadvantage of this method is the need to have a reactor equipped with the appropriate infrastructure available.

### 4.1. The Hydrogen Problem

There are many data on impurities in DND and their structure, including the impurity of hydrogen atoms. The hydrogen content in the untreated powder of DND particles can reach 4.8% by weight [114]. Hydrogen is contained in DND both in the form of a chemical bond with carbon in the CH, CH_2_, or C-OH functional groups and in the form of adsorbed water from the air on the sp^2^ carbon shell, which is located on the diamond core. This shell contains hydrophilic –OH and –COOH functional groups, which favor water adsorption through hydrogen bonds [5]. In addition, as was found out during the preparation of the experiment described in Section 3, the powder has a high hygroscopicity, which is explained by the adsorption of water. Two types of water adsorption can be distinguished: physisorption and chemisorption.

There are a wide range of studies devoted to the DND hygroscopicity (see, for example, the review [114]). Thus, in ref. [115] the isotherms of water adsorption by DND were shown and a model of primary adsorption centers was proposed. The authors stated that water adsorption begins with the interaction of water molecules with these centers. In ref. [116], DND water adsorption was studied using Fourier transform infrared spectroscopy, and, based on the results, three forms of adsorbed water were proposed. As a result of the experiments and modeling, the authors of [115] explained the adsorption of water by surface functional groups and porosity (micropores, mesopores) of DND powders of detonation synthesis. ND aggregates have a pore mouth structure that allows for the smooth access of water molecules [116].

**Figure 13:**
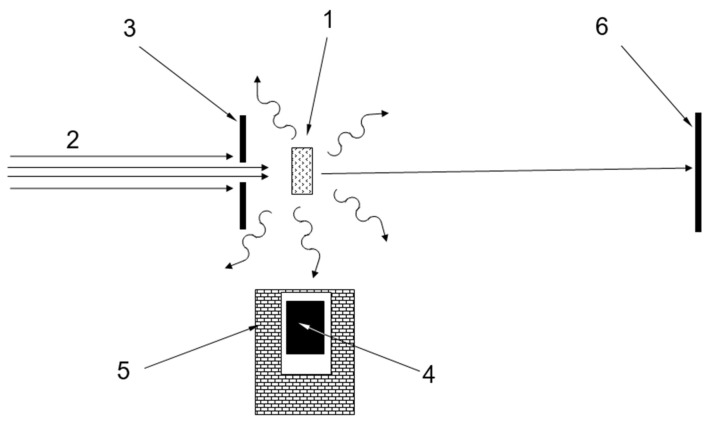
A scheme for measuring the hydrogen content in powders. (1)—sample; (2)—neutron beam; (3)—collimator; (4)—germanium γ -detector; (5)— γ and neutron protection; (6)—monitor neutron detector. A similar figure is given in ref. [97].

**Figure 14:**
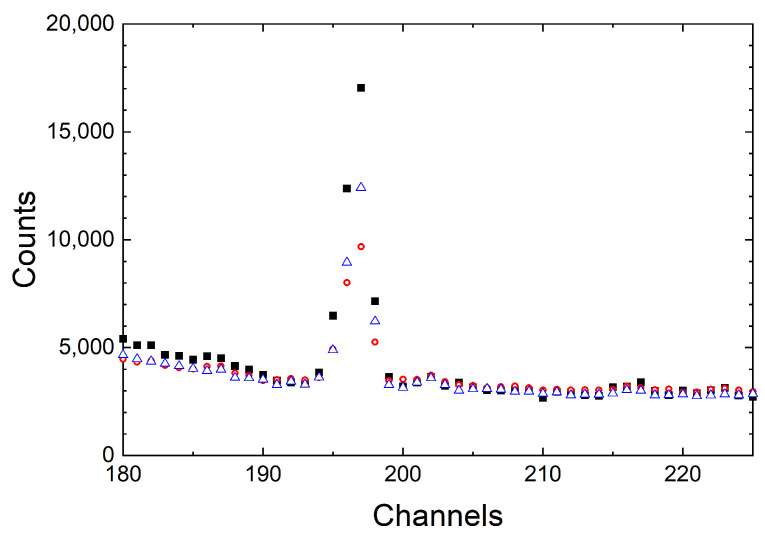
The peak of total absorption in the reaction n(pd)γ for polyethylene (squares), for Sample 1 (circuits) and Sample 2 (triangles) (see Table 2). Spectra from an aluminum sample with a thickness equal to that of the aluminum containers are subtracted from the spectra measured with nanopowders. A similar figure is given in ref. [97].

The absolute content of hydrogen in the DND powder used in the experiment [65] was measured via NAA using prompt γ -quanta ( $E_{\gamma }=2.223$ MeV). A measurement scheme [97] is shown in Figure 13, a typical spectrum in Figure 14, and the results in Table 2.

The following ratios of carbon to hydrogen in the samples were obtained:

$n_{C}/n_{H}=12.4\pm 0.2\;\left(C_{12.4\pm 0.2}H\right)$ for the sample purified from H via heating in a vacuum and

$n_{C}/n_{H}=7.4\pm 0.15\;\left(C_{7.4\pm 0.15}H\right)$ for the sample not purified from H.

Inelastic neutron scattering measurements showed that the hydrogen that cannot be removed through pumping or heating is chemically bound to carbon [97]. The same powder was studied as in [65,67].

A large number of attempts have been made to obtain NDs without hydrogen. Often, they led to the disappearance of the diamond form of carbon in the powders. Most of them turned out to be unsuccessful. In some cases, the amount of hydrogen could be reduced [117] but was still large.

This direction of research competed with the searches for methods of substituting the hydrogen, which turned out to be successful. To purify DND from impurities of hydrogen atoms and to increase the efficiency of neutron reflection from DND, chemical cleaning to DND was applied [69]: gas (F_2_)–solid fluorination. Such a chemical treatment led to a more than thirty-fold decrease in the amount of chemically bound hydrogen, which was measured using the instantaneous γ -quanta in the n(p,d)γ reaction. The ratio of carbon and hydrogen corresponds to the formula $C_{430\pm 30}H\left(n_{C}/n_{H}=430\pm 30\right)$ .

The procedure for the gas–solid fluorination of DND powder was carried out in two stages under static conditions in a nickel reactor. DNDs were placed on passivated nickel substrates (coated with NiF_2_) inside the reaction vessel (passivated with NiF_2_ too). Before the introduction of gaseous molecular fluorine (F_2_), the reactor was evacuated to a vacuum of ∼ $10^{-2}$ mbar for 12 h. Then, pure fluorine gas (purity 99.9% ) was added to achieve a pressure of 0.6 atm inside the reactor. The pressure was chosen in order to avoid exceeding the pressure of more than 1.2 atm during heating at 450 °C and the release of decomposition gases as a result of fluorination.

The fluorination procedure with fluorine gas was carried out at a temperature of 450 °C for 12 h. After the first stage and cooling to room temperature, the reactor was purged with a flow of nitrogen for an hour to remove unused fluorine, HF, and decomposition products (CF_4_ and C_2_F_6_). As a result of the procedure, the chemically bonded hydrogen in the CH, CH_2_, and C-OH groups was replaced by fluorine atoms. Moreover, the sp^2^ shell on the diamond surface was removed via a decomposition reaction into CF_4_ and C_2_F_6_ gaseous species. In other terms, this shell burned in a pure fluorine atmosphere. The decrease in the number of sp^2^ atoms and hydrogen-containing groups was confirmed through measurements using IR, Raman and nuclear magnetic resonance spectroscopies.

The neutron absorption cross-section of fluorine ( $\sigma_{a}^{F}=9.6$ mb) is much smaller than the hydrogen absorption cross-section $\sigma_{a}^{H}$ , and the coherent elastic scattering length is large and positive ( $b_{F}=5.65$ fm) and differs largely from the negative scattering length for hydrogen ( $b_{H}=-3.74$ fm). Thus, all the key properties of fluorine correspond to the requirements for materials for effective nanopowder neutron reflectors.

Thus, the problem of hydrogen impurities was solved. Moreover, the lower hygroscopicity and, accordingly, the greater stability of the properties of the powder associated with the accumulation of hydrogen is an additional advantage of fluorinated DND (F-DND). Adsorption sites for water molecules are removed and/or replaced by hydrophobic C-F bonds.

Despite the general solution of the hydrogen problem by replacing hydrogen atoms with fluorine, the question still existed about the stability of F-DND to reverse substitution with hydrogen. The long-term hydrophobic properties of F-DND were tested using gravimetric measurements. After 3 years of storage of an F-DND sample in the atmosphere, the ratio of hydrogen and carbon deteriorated by about 12 times. Moreover, this is probably hydrogen rigidly bonded to diamond as the measurements were carried out in a vacuum.

The appearance of hydrogen in the powder was confirmed through measurements on the infrared spectrometer, which showed the formation of C-OH and C-H bonds, as well as through neutron prompt- γ analysis. A repeated fluorination procedure led to the disappearance of the signal from these bonds.

Because of the metastability of diamond, especially with a nanometric size, the rebuilding of the diamond surface may be at the origin of the reappearance of a sp^2^ shell and consequently H atoms.

While the fluorination process basically solves the problem of hydrogen contamination, one needs to follow certain rules when storing the powder to exclude the reverse substitution of fluorine for hydrogen over a long time and to finally establish the stability of fluorine at high radiation fluxes.

### 4.2. The Problem of Metals

Ref. [69] presents the results of measuring impurities using the NAA method for a powder before treatment; after fluorination; after fluorination and additional purification; and after fluorination and twice-repeated additional purification. They are shown in Table 3. In addition to the elements listed in the table, the following elements were found in amounts less than 1 ppm: Co, As, Mo, Sb, Hf, W, and Au. The following elements were found in amounts less than 0.1 ppm: Se, Rb, Zr, Ru, Ag, Sn, Cs, La, Ce, Pr, Nd, Sm, Tb, Ho, Tm, Yb, Lu, Ta, Re, Ir, Os, Pt, and Hg. Additional purification consisted of treatment with concentrated hydrochloric acid at the temperature of 140 °C for 18 h, followed by washing and drying.

Fluorination with pure elemental fluorine of the impurities at a high temperature (450 °C) results in most of the cases in the formation of non-volatile fluorides with the highest oxidation state for the metal, e.g., NaF, KF, MnF_3_, FeF_3_, CuF_2_, ZnF_2_, and BaF_2_. There are some special cases for which volatile species are formed: ClF_3_, BrF_3_, SiF_4_, AsF_5_, and PtF_6_. Metal impurities must be removed using another chemical route before or after the fluorination.

As is clear from Table 3, the fluorination procedure had no effect on impurities other than hydrogen, while the purification significantly reduced the content of metals (Cr, Cu, Zn). It can be seen that metal impurities from chemical purification can be brought to a state where the loss of neutrons on them becomes small compared to the capture on nitrogen (see Section 4.3) and carbon itself. Similar results were obtained in the processing of DND powder made from another explosive (tetryl) by another manufacturer and purified using a different technology. It was confirmed that hydrochloric acid is a very effective reagent for cleaning powder from metals; however, from Table 3 and from other experiments, it can be seen that after the use of hydrochloric acid, the chlorine content increases. Chlorine itself is an important neutron absorber, so purification technologies require further development.

Despite the insignificant amount, from the point of view of neutron capture, of metal impurities in the powder, it must be borne in mind that even a small amount of metals in intense neutron fields can lead to noticeable activation. Considering that the mass of powder in a real reflector must be quite large (of the order of kilograms), this will lead to the appearance of noticeable activity at the reflector. Since the DND powder is quite volatile, it can be difficult to work with the activated powder. In this case, elements whose impurities are less than ppm but which have relatively long decay periods after activation, for example, and ^182^ Ta (half-life 115 days) ^60^ Co (half-life 5.27 years), may turn out to be significant. This issue has not yet been studied in detail, but it may turn out that the design of reflectors will require scaling up other technologies for the production of DND, which allows DND without metal and nitrogen impurities to be obtained.

### 4.3. The Problem of Non-Metallic Absorbers (Cl, B, N)

One should note the following important impurities, from the point of view of neutron losses: nitrogen, possibly boron (it is poorly determined by NAA), and chlorine (arising in particular from the purification of powders from metals). Nitrogen is located inside diamond crystals and it is difficult to remove it without changing the DND production technology. In the measurements described above, a noticeable amount (up to 30 ppm) of boron was detected; however, quantitative evaluations are not reliable because of the large background associated with a significant signal from the neutron shield containing boron. Boron is a fairly common element, and even its slight admixture (tens of ppm) could noticeably change the properties of the DND powder because of the large ( 3835b [118]) capture cross-section of the ^10^B isotope, which is present in a natural mixture in a noticeable amount ( 20% ). Thus, during the production, purification, and processing of DND, care must be taken not to introduce boron as an impurity into the final product.

## 5. Studies of the Effect of ND Powder Parameters on Reflection

An experiment on the precision measurement of the albedo from DND requires a significant amount of powder. At the same time, manipulations with DND powder (purification from impurities, fluorination, changing the structure of the powder) are available in laboratories with small (on the order of a gram) amounts of material. In refs. [20,73,76], a method was developed that allows for the characterization of a powder using a small amount of it and the calculation the albedo from the large sample of such a powder in any configuration.

One of the main principles of operation of DND-based neutron reflectors is the fact that, upon shaping separate atoms into a nanoparticle, the coherent scattering of neutrons by nanoparticle atoms is greatly enhanced, while inelastic scattering remains the same [87]. Consequently, DND neutron scattering is mainly elastic, and the atomic dynamics of the system does not play a big role. As a result, the scattering and transport of neutrons in DND powders are determined by the properties and relative position of DND particles, rather than by the strength of the physical and chemical bonds between them. The smallest object is a DND primary particle with a diamond-density core. DND particles can be combined into clusters and agglomerates. Here and below, the distinction between agglomerates and clusters is implied. Clusters are called areas of denser DND packing, since only such objects are relevant to neutron scattering. In the zero approximation, an important parameter is the average bulk density of the cluster. The properties of clusters, which are important for diffusion and neutron transport, may not coincide with the properties estimated by other methods. Agglomerates are large associations of clusters in which these clusters are weakly interconnected.

The accurate determination of the nanoparticle size distribution in a powder, including the description of their clustering and agglomeration for the subsequent calculation of neutron scattering on this structure, is a difficult task both from the point of view of determining the parameters of the powder and from the point of view of the scattering description. For example, experimental methods that allow for the obtainment of information about the sizes of crystals and their agglomerates give a different picture due to the different physical principles underlying them.

Various methods can be used to measure the sizes of DNDs and their clusters in DND powders. These methods are widely used in both materials science and neutron scattering. These include small-angle neutron scattering (SANS) and X-ray scattering (SAXS), the broadenning of the peaks in neutron diffraction and X-ray diffraction (XRD), neutron quasi-specular reflection (QSO), TEM, scanning electron microscopy (SEM), dynamic light scattering (DLS), etc. All these methods allow for the obtainment of fundamentally different information and can be used to solve specific problems.

SANS provides the most direct information for the analysis of the scattering and transport of VCNs and CNs in DND powders. The method is sensitive both to individual DNDs and to their clusters. Since the probability and angular characteristics of scattering depend on the neutron velocity, in order to apply the measurement results to neutrons of other velocities and other scattering angles, it is necessary to use a mathematical model describing the scattering medium.

SAXS is sensitive to the electronic structure of matter and not to the neutron optical potential (like SANS). In particular, the contribution of hydrogen atoms to SAXS is much smaller than to SANS, while the contribution of heavy metal impurities is relatively higher. However, in the case of DND powders, SAXS and SANS give similar results, since scattering is determined by carbon atoms, while other impurities are small.

The methods of diffraction and small-angle scattering of neutrons and X-rays complement each other and provide detailed information about the scattering properties of powder samples.

Neutron diffraction and XRD provide similar information and are sensitive to the crystal structure of diamond cores of nanoparticles. These methods are insensitive to sp^2^ shells and DND clusters.

The QSR on a reflectometer provides information obtained by all the indicated methods. However, multiple neutron scattering, which is important in the implementation of this method, requires the use of different models to analyze the results.

**Figure 15:**
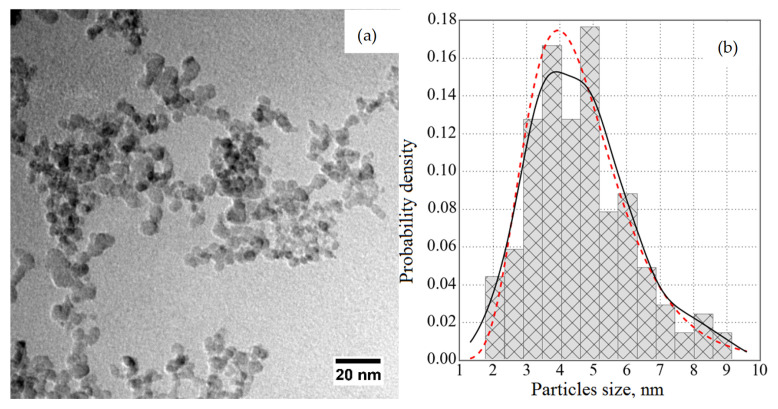
(**a**) TEM image of an F-DND sample; (**b**) DND diameter distribution calculated from a set of similar images. The red dotted line corresponds to the lognormal distribution. The black solid line indicates the result of fitting the data with a smooth dependence. The figure is copied from ref. [73].

**Figure 16:**
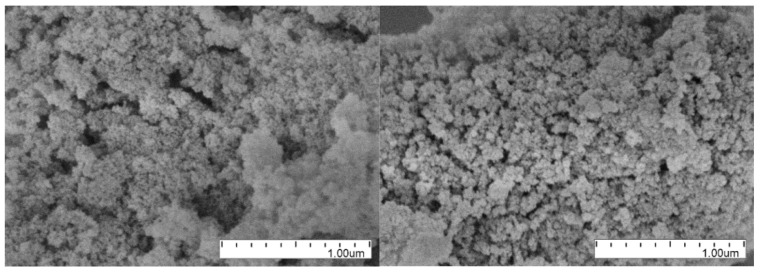
Examples of SEM images of DND (**left**) and F-DND (**right**). The figure is copied from ref. [73].

TEM and SEM serve to visualize the powder structure. These methods make it possible to estimate both the sizes of DNDs (including sp^2^ shells) and the sizes and structure of clusters. However, the exact determination of the size distribution is more difficult than using neutron or X-ray scattering methods, since it requires the analysis of a large number of images. Examples of TEM and SEM [73] images are shown in Figure 15 and Figure 16, respectively.

The DLS method is less effective for studying DNDs as neutron reflectors, since it studies a fundamentally different phenomenon. It is sensitive to the motion of individual DND particles and their agglomerates as a whole in liquid dispersions. In addition, DND agglomerates in liquid dispersions and in initial dry powders are different [119]. The size of the DND obtained through this method is overestimated because it includes layers of water entrained by the DND particle as it moves. Even if there is only a small proportion of large particles, the resulting average size may appear larger than the real one. Moreover, hydrophobic F-DND cannot be studied in water dispersion.

**Figure 17:**
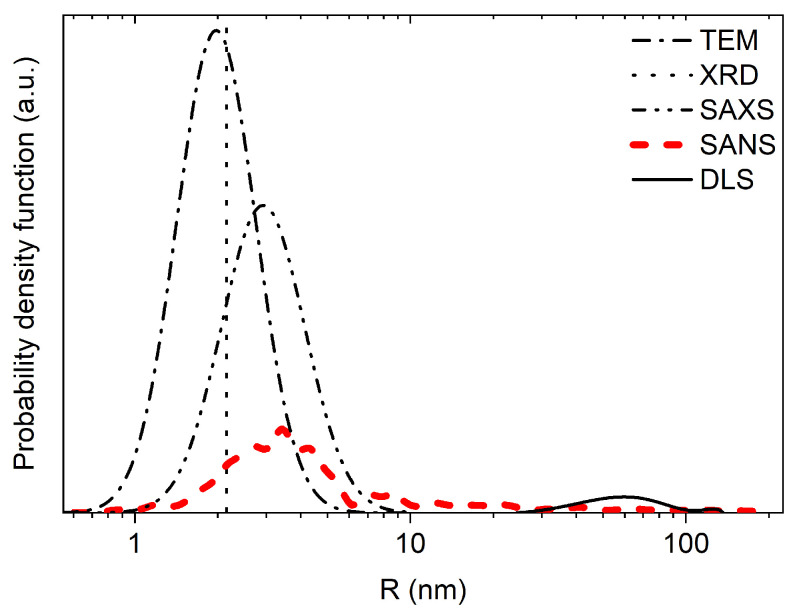
The probability density of the size distribution of scatterers as a function of their radius [nm] estimated using SANS (thick red dashed line), SAXS (thin dotted dash–two-dotted line), TEM (black thin dash–dotted line), and DLS (black thin solid line). The vertical black dotted line indicates the median radius as measured via radiography. All measurements were performed with F-DND samples, with the exception of SAXS, which was performed with a DND sample. A similar figure is given in ref. [73].

As an illustration of the fact that different methods can provide different information when measuring the same quantity, Figure 17 shows the probability densities of the size distribution of neutron scatterers in powder, obtained by various methods (SAXS, SANS, XRD, TEM, and DLS) [73].

Comparison of the distributions presented in Figure 17 allows us to conclude that the results obtained via the SANS, SAXS, XRD, and TEM methods give consistent results. The fact that SANS and SAXS give slightly larger sizes may be due to the fact that in these methods two or more closely spaced DND particles can produce almost the same scattering curves as one larger DND [105]. At the same time, the differences between the estimates of the average size of scatterers obtained via all these methods (SANS, SAXS, XRD, TEM) and the estimate of the average size from the DLS results are an order of magnitude. This is due to the fact that, as already mentioned above, they characterize different details of the DND structure. The value of 50–100 nm, measured using DLS, is typical for the size of agglomerates [120].

The idea of the method mentioned at the beginning of this section is to measure the absolute SANS cross-section using a small sample as a function of the transferred momentum and then describe the result via neutron scattering on a model medium. The model parameters are determined by fitting them to describe the experimental cross-section. Further, this simulation is used to model the neutron transport. Losses due to neutron capture are modeled based on data on the elemental composition of the powder.

SANS can be performed on a standard instrument using CNs or even thermal neutrons, since the dominant Bragg scattering effect scatters thermal neutrons at large angles. Absolutization of the cross-section for SANS for thermal neutrons is difficult.

A model of diamond nanoballs with a discrete size distribution is proposed as a model medium. Both DND particles and their clusters in this model are replaced by diamond nanoballs of various sizes. A discrete set of nanoball sizes was chosen in which the radii of any nanoball of the next size are related by a certain ratio. For example, radii are uniformly distributed on a logarithmic scale, with 20 radius values per order of magnitude.

Populations of nanoballs of each size are selected in such a way as to best describe the experimental data. The fitting procedure is stable with respect to the choice of model parameters (initial populations of nanoball sizes; nanoball size increase step; size range limits, if it is wide enough).

When analyzing the resulting size distribution of diamond nanoballs, it should be taken into account that actually existing clusters differ significantly in density from the density of diamond and have a shape that does not coincide with an ideal ball. The neutron scattering cross-section for a real cluster is smaller than the scattering cross-section for an ideal diamond nanoball. Consequently, the number of clusters is greater than that obtained in our model as many times as the scattering cross-section is larger. However, for calculating the diffusion of neutrons in DND powders, the diamond nanoball model gives a good approximation. Accordingly, the total mass concentrated in diamond nanoballs, estimated within our model, is lower than the total mass of the powder. This feature effectively takes into account (i) the presence of carbon in non-diamond forms (sp^2^) in the powder; (ii) elements other than carbon; (iii) pore scattering; (iv) the difference between the shapes of DND diamond cores and ideal nanoballs; (v) scattering interference from neighboring DND particles, etc.

In a sense, this simple clustering model is analogous to the expansion of a mathematical function in a series into simple basis functions. The amplitude of each expansion term does not directly reflect the physical reality but allows us to analyze the general behavior of the function under study. The effective weight of the powder, thus estimated, is a useful characteristic. It gives the mass fraction of the powder, which is effectively involved in neutron scattering. The remaining mass effectively participates in the loss of neutrons but does not participate in scattering. The larger the effective mass, the higher the efficiency of the DND powder for reflecting neutrons.

**Figure 18:**
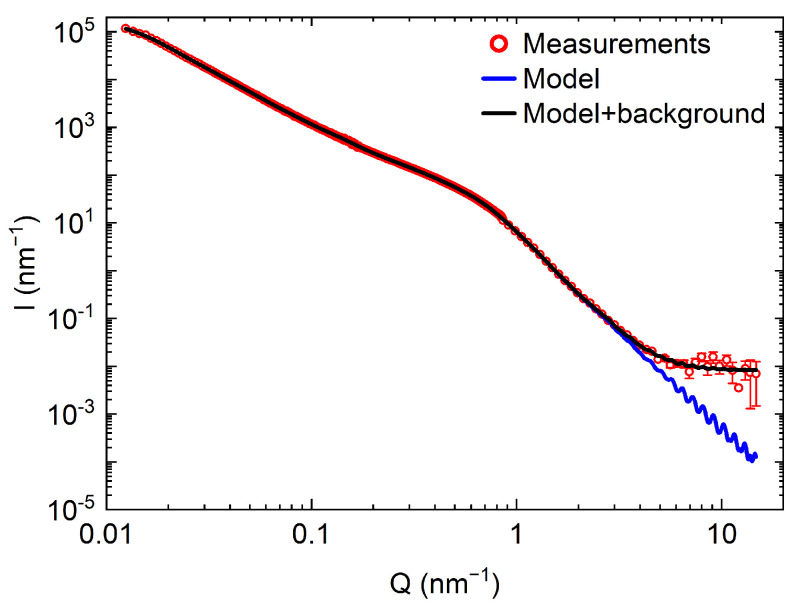
Comparison of the measured and simulated intensity *I* (cm^−1^) of scattered neutrons as a function of the transferred momentum *Q* (nm^−1^) for an F-DND sample. Black squares represent experimental data. The thin blue line shows the results of modeling within the model of discrete-size diamond nanoballs, and the thick red line additionally takes into account the background contribution at the level of $9\times 10^{-3}$ nm^−1^. A similar figure is given in ref. [73].

**Figure 19:**
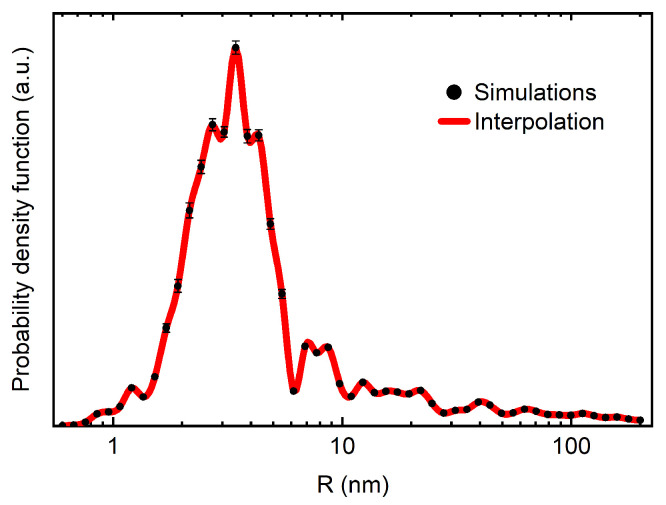
Probability density for the distribution function of nanoballs as a function of radius (in nm), estimated within the model of diamond nanoballs of a discrete set of sizes for the F-DND sample. Black circles show simulation results. The red solid line interpolates the simulation results. A similar figure is given in ref. [73].

Using this approach, ref. [73] showed that the fluorination process did not affect the scattering power of the powder. Based on the dependences of the intensity of SANS on the magnitude of the transferred momentum (Figure 18), the distributions of the nanoballs (Figure 19) in the model medium were constructed and the intensities of SANS were modeled (Figure 18).

The mass distribution of nanoballs in Figure 19 contains, as shown, a population of DND particles and a population of DND clusters (which are associated with larger nanoballs) of different sizes. Although the mass fraction contained in large clusters is relatively small, they determine the scattering of neutrons in the range of the small transferred momentum *Q*, as shown in Figure 18.

The results obtained show that DND and F-DND powders are identical in the range of structure radii from 0.6 nm to 200 nm. The maximum agglomerate sizes observed in the present measurements are in the agglutinate size range. Thus, we can conclude that, for SANS, fluorination does not change the size of individual DND particles or the size and structure of primary agglomerates.

The actual lack of effect of fluorination on the scattering properties of DND was not apparent prior to the present experimental study and therefore deserves discussion. Certain information is known about changes in the composition of DND during fluorination. The DND mass remains almost unchanged, while hydrogen and the amorphous DND sp^2^ shell are almost completely eliminated [69]. Fluorination at least partially removes another important impurity, namely oxygen, whose fraction can reach 10% [8]. The fraction of nitrogen may decrease (because of the evolution of NF_3_ gas during the fluorination, if N is accessible for F_2_), but it has not been studied quantitatively. NAA showed that the composition and amount of metal impurities in powders did not noticeably change [69].

**Figure 20:**
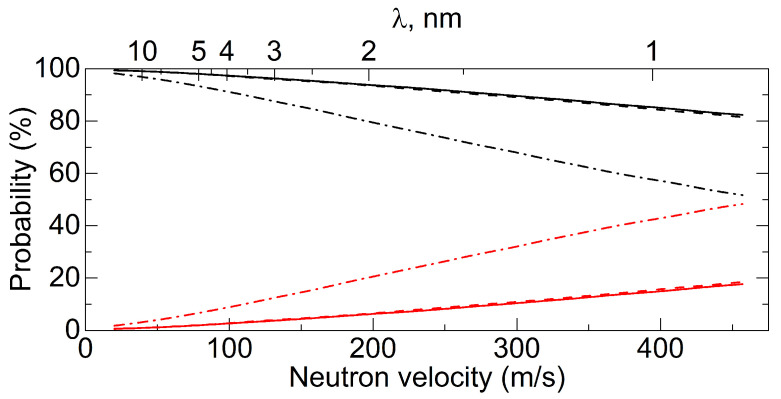
Probability of neutron reflection (black lines) and absorption (red lines) for F-DND (solid lines) and DND (dash-dotted lines) depending on neutron velocity (on bottom) and wavelength (on top). The incident neutron flux is isotropic, the density of the powder is 0.19 g/cm^3^, and the thickness is infinite. A similar figure is given in ref. [20].

The change in the albedo in this case is due to a significant decrease in losses associated with the capture of neutrons by hydrogen atoms. For a model medium with the distribution of nanoballs shown in Figure 19, the Monte Carlo method was used to calculate the albedo from a semi-infinite medium of DND and F-DND powder. Figure 20 shows both the reflection probability and the corresponding absorption probability. For the DND powder, the capture on hydrogen is taken into account; for F-DND, the losses are associated only with the capture by carbon atoms.

In a similar way, the effect of (F-)DND deagglomeration [20] and changes in the size of nanoparticles [76] on the albedo of VCNs were studied.

The deagglomeration process was studied on an F-DND powder. It significantly decreased clusters, which is confirmed by the results of DLS measurements. This method is sensitive to the movement of DND particles and agglomerates in a liquid dispersion. One should note that the clustering of DND particles immersed in a liquid may differ from clustering in a dry powder. In addition, DND clustering in a dry powder after deagglomeration may differ from that measured in a liquid after deagglomeration.

**Figure 21:**
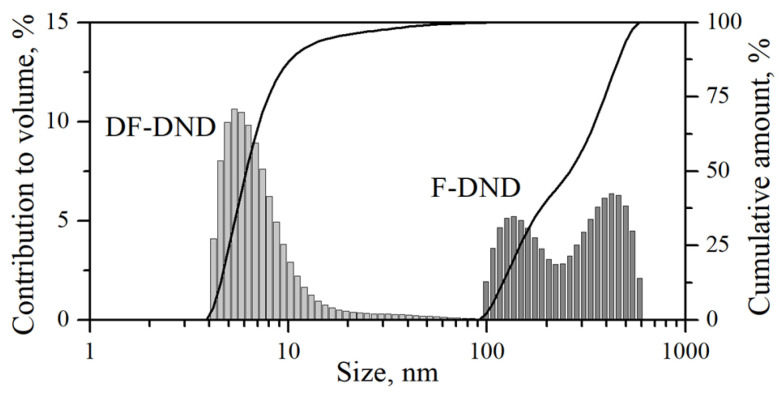
Size distributions of F-DNDs and DF-DNDs in ethanol, measured using the DLS method. The figure is copied from ref. [20].

The size distribution of F-DND and DF-DND (deagglomerated F-DND) dispersed in ethanol was measured via DLS. F-DNDs mainly form agglomerates with sizes of 100–600 nm (F-DNDs in Figure 21). The agglomerates have a bimodal size distribution with maxima at 130 nm and 420 nm. The centrifugation of the colloidal solution with F-DND made it possible to precipitate most of the agglomerates. Individual 5.5 nm F-DNDs were observed via DLS in the supernatant extracted after centrifugation (DF-DNDs in Figure 21). It should be emphasized that the F-DND suspension prepared via strong ultrasound contains deagglomerated particles, but the presence of agglomerates does not allow them to be detected via DLS [121]. Individual particles could only be detected after removing most of the agglomerates from the suspension.

**Figure 22:**
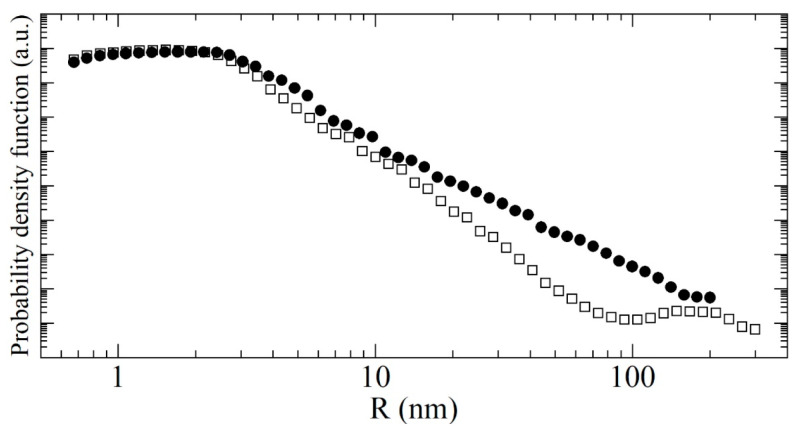
Probability density as a function of radius (in nm) for the size distribution of nanoballs, calculated in the model of discrete-size diamond nanoballs for F-DND (solid circles) and DF-DND (open squares). The points correspond to the simulation. The figure is copied from ref. [20].

A decrease in the number of aggregates was also observed in the simulation. Figure 22 shows a double logarithmic plot of the distribution of scatterer radii in both samples, extracted from the simulation of the SANS data. As can be seen from these graphs, small radii dominate the distribution, and their number does not change significantly during deagglomeration. The decrease in the proportion of large clusters is clearly visible. The fraction of these clusters in the distribution is not large, but neutron scattering by them dominates in the region of low momentum transfer.

This change in the powder structure did not significantly affect the albedo from the semi-infinite medium. However, the deagglomeration process significantly increased the bulk density of the powder. It increased from 0.19 g/cm^3^ to 0.56 g/cm^3^. This increase becomes significant if the possible thickness of the reflector is limited or the reflector has an infinite thickness but has the shape of, say, a spherical cavity. In both cases, an additional parameter arises (thickness, cavity size) with which it should be compared to the neutron transport cross-section in the medium, and it depends on the density.

**Figure 23:**
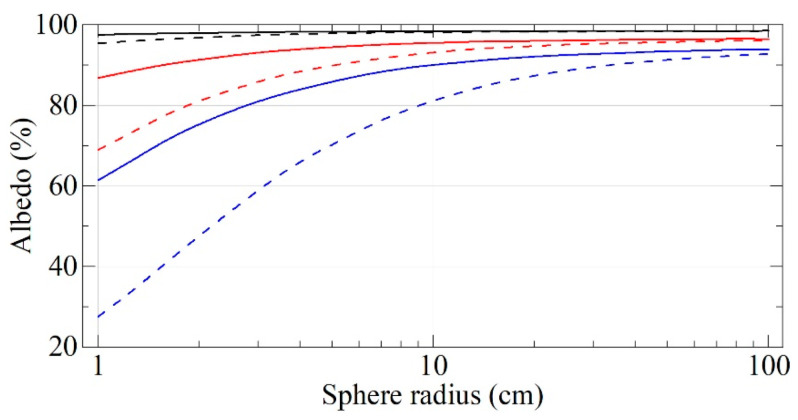
Neutron albedo for VCN velocities of 50 m/s (black lines), 100 m/s (red lines), and 150 m/s (blue lines) for F-DND (dashed lines) and DF-DND (solid lines) depending on the cavity radius. The incident neutron flux is isotropic, the powder thickness is infinite, and the densities of the DF-DND and F-DND are 0.19 g/cm^3^ and 0.56 g/cm^3^, respectively. The figure is copied from ref. [20].

Figure 23 shows the albedo of neutrons from the walls of the cavity as a function of the neutron velocity and radius of the cavity for both the F-DND and DF-DND powders. In both cases, the real bulk density was taken in the calculation. A higher albedo means a higher neutron density that can be stored in such a cavity.

In the considered geometry, DF-DNDs show much higher albedo values compared to F-DNDs in the entire range of neutron velocities for all values of the cavity size. Moreover, the gain reaches the greatest value in the case of an approximate equality of the cavity radius and the depth of penetration of neutrons into the powder upon reflection.

**Figure 24:**
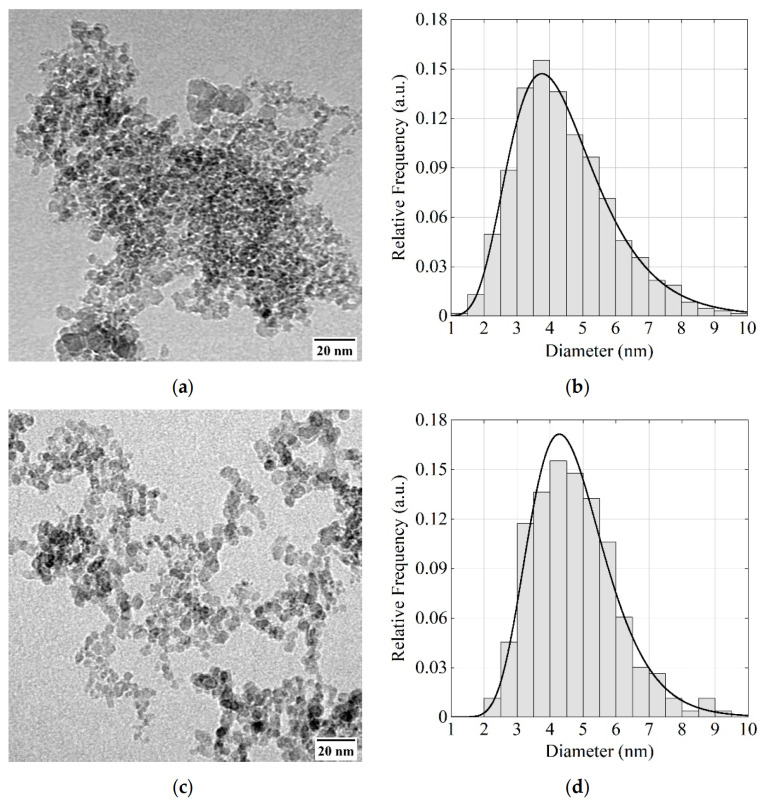
(**a**,**c**) Example TEM images of DF-DND and S-DND; (**b**,**d**) aize distributions for DF-DND and S-DND estimated using all available TEM images. The figure is copied from ref. [76].

**Figure 25:**
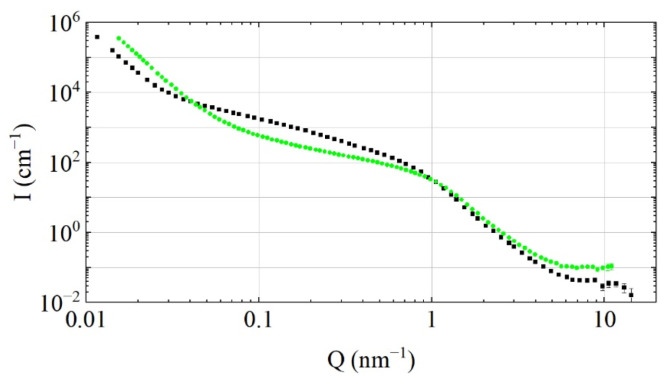
Neutron scattering intensity *I* (cm^−1^) versus momentum transfer *Q* (nm^−1^) for DF-DND (black squares) and S-DND (green circles). To compare only the effect of particle size and not powder density, both curves are normalized to the sample density of 1 g/cm^3^. The figure is copied from ref. [76].

When studying the effect of changing the size of nanoparticles [76] on the albedo of VCNs, a fluorinated deagglomerated powder and a powder obtained via the separation of the starting material after deagglomeration but before fluorination were compared. The differences in the sizes of nanoparticles in powders were shown using various methods: TEM (Figure 24), XRD, and SANS (Figure 25). In addition to changing the size, the separation process further increased the bulk density of the powder, which reached 0.67 g/cm^3^.

**Figure 26:**
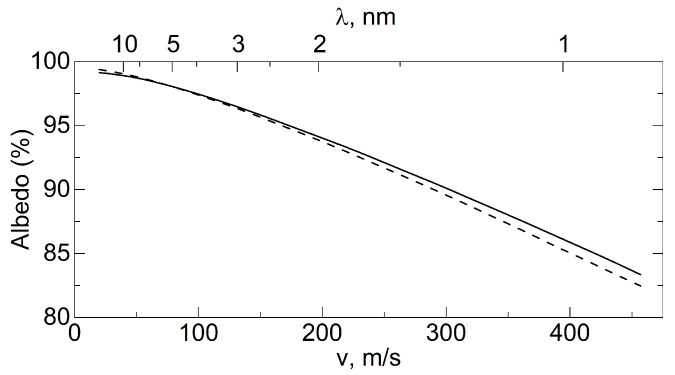
Neutron albedo for flat semi-infinite media DF-DND (dashed lines) and S-DND (solid lines) depending on the neutron velocity and wavelength. A similar figure is given in ref. [76].

Figure 26 shows the calculated neutron albedo from a semi-infinite medium for DF-DND and S-DND powders; small DND (S-DND) particles were separated from DND powder using the method of centrifugation, as described in ref. [76]. At VCN velocities <70 m/s, the albedo is higher for DF-DND, while at VCN velocities >70 m/s it is higher for S-DND. This is due to the presence of large particles in DF-DND, which are closer to the optimal values for lower velocities and, conversely, the presence of smaller particles in S-DND, which are closer to the optimal values for higher velocities (See Figure 2).

**Figure 27:**
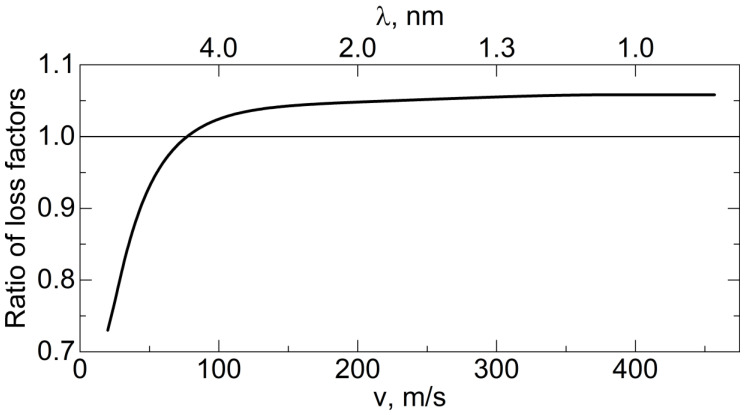
Ratio of loss coefficients $\eta_{DF-DND}/\eta_{S-DND}$ upon reflection from flat semi-infinite media of DF-DND and S-DND depending on the neutron velocity. The corresponding neutron albedo is shown in Figure 26. A similar figure is given in ref. [76].

The relatively small difference between these absolute albedo values translates into a large difference between the loss factors ( η=1−albedo ), especially for albedo values close to 100% . Since the VCN densities that can accumulate in a trap with DND walls are inversely proportional to the loss factor, the results shown in Figure 26 were used to calculate the loss factor ratio shown in Figure 27.

Figure 27 shows that DF-DNDs are more advantageous than S-DNDs for reflecting neutrons with typical VCN velocities ∼50 m/s from semi-infinite flat media. However, when the goal is to increase the upper edge of the VCN velocity range, S-DNDs are somewhat better suited than DF-DNDs.

**Figure 28:**
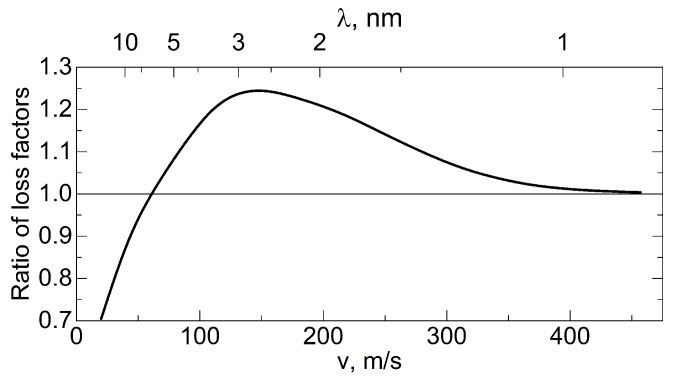
The ratio of loss coefficients $\eta_{DF-DND}/\eta_{S-DND}$ upon reflection from the wall of a spherical cavity with a radius of 5 cm and a wall thickness of 3 cm, for realistic powder densities of 0.56 g/cm^3^ for DF-DND and 0.67 g/cm^3^ for S-DND. A similar figure is given in ref. [76].

An increase in density again leads to an increase in the albedo in the case of a reflector of finite thickness or for a spherical trap. A comparison of the characteristics of reflectors based on DF-DND and S-DND for a realistic geometry of a spherical cavity with a radius of 5 cm and a wall thickness of 3 cm for realistic powder densities is shown in Figure 28.

Comparison of Figure 26 and Figure 28 evidences that (1) S-DNDs are more beneficial for reflecting high-velocity VCNs starting from ∼60 m/s; (2) the decrease in the loss factor for S-DNDs is ∼20–25% in a wide range of VCN velocities. The first point is important when estimating the VCN spectrum returning from the DND reflector. The second factor is directly converted into an increase in the VCN density, which can be accumulated in traps with DND walls when S-DND and DF-DND powders are used. The relative decrease in the gain coefficient with an increasing velocity in Figure 28 is the result of the partial passage of faster neutrons through a wall of finite thickness.

As Formula (13) shows, the optimal DND diameter for VCNs with a velocity of ∼50 m/s is ∼ 4.3 nm. If the goal is to increase the upper limit of the range of effective reflection rates of VCNs, then the average diameter of the DND should be reduced. However, it cannot be reduced by more than about a factor of 3, since even smaller DNDs do not exist. In addition, the cross-section for coherent neutron scattering sharply decreases with a decreasing DND diameter (as the sixth power of the diameter [87]).

If the goal is to increase the storage time for the softer part of the VCN spectrum, the average DND diameter should be increased. Increasing the particle size by more than 3 times makes no sense, since larger nanoparticles are effective for neutrons with velocities in the UCN range, for which there are other effective reflection mechanisms (optical neutron–nuclei Fermi potential and supermirrors). These arguments approximately determine the range of effective reflection of VCNs from DND and the choice of DND parameters for the design of DND reflectors optimized for the diffuse reflection of VCNs.

## 6. Projects to Use ND Reflectors to Enhance VCN and CN Fluxes

We have separated this topic into a separate short section only because of its importance. The fact is that any new method is of interest only if it is actively used in practice. At the moment, the applicability of the method of nanodiamond reflectors of slow neutrons has already been convincingly proven; some specific projects have already been proposed [32,78,79], but they have not yet been implemented.

There are at least four reasons for this. Firstly, reflectors with parameters close to optimal were designed quite recently [20,69,73,76,78,80]. Secondly, such reflectors with optimal parameters are still produced in laboratory quantities, and methods for upscaling the production of optimal powders still need to be developed. Thirdly, the presence of impurities in powders leads to the fact that the powders can be activated in large radiation fluxes, which will complicate their manipulation; It would be useful to learn how to clean nanodiamond powders in advance. Fourth, the stability of diamond nanoparticle cores in strong radiation fluxes has not yet been sufficiently studied. DNDs are metastable nanomaterials, which are temporarily stabilized by fluorination and the presence of C-F bonds on their surface. Their stability under high neutrons fluxes must be investigated.

Therefore, for now we will limit ourselves to only a general statement that the first specific projects for the use of nanodiamond reflectors are emerging, and the implementation of at least one of them is the most interesting continuation of this activity.

## 7. Conclusions

We reviewed the new field of research, methods, and applications associated with nanodiamond slow neutron reflectors. At present, the principle of operation of such reflectors has been convincingly proven. On the one hand, reflectors based on fluorinated nanodiamond powders provide record efficiency in reflecting slow neutrons. Moreover, the reflection efficiency can be improved by eliminating impurities that lead to neutron loss, as well as by optimizing powder parameters. For practically interesting cases, the reflection efficiencies do not seem to be very far from the best theoretical values. On the other hand, for the practical application of nanodiamond reflectors, several issues still need to be resolved and/or investigated. The problem of the deep purification of nanodiamond powders from impurities that can be activated in strong radiation fluxes has not yet been solved. Its solution would reduce the difficulty of manipulating such activated powders. In addition, the stability of diamond nanoparticles in high radiation fluxes has not been sufficiently studied. Finally, fluorinated nanodiamond powders with optimized parameters are still produced in laboratory quantities, and upscaling methods for preparing such powders require implementation. After solving these problems, the active use of the nanodiamond reflector method will have high prospects in neutron science and technology.

## 8. Patents

V. Nesvijevski, A. Bosak, “Procede de fabrication d’un reflecteur de neutrons et reflecteur de neutrons obtenu par un tel procede”, 2014, Regimbeau, France. 1463376.

## Figures and Tables

**Table 1:** Parameters of some materials that can be considered for nanoparticle reflectors.

Nanoparticle Material	Volume Density, g/cm^3^	V_0_, neV	V_1_, $10^{-6}$ neV
D_2_	0.195	101	2.2
D_2_O	1.02	153	2.7
O_2_	1.23	70	0.64
CO_2_	1.56	101	6.0
C (diamond)	3.52	306	45
Be	1.85	250	68

**Table 2:** Characteristics of the samples used in the measurements of the hydrogen content.

Sample	Thickness, d [mm]	Density, ρ [g/cm^3^]
Polyethelene	1.5	0.91
Sample 1 (DND powder after purification from H at 150 °C under pumping for 23 h)	40	0.378
Sample 2 (DND powder without preliminary heat treatment)	40	0.300

**Table 3:** The content of impurities after fluorination and purification in the DND samples (according to ref. [69]).

Element	$\sigma_{a}$ , b	Before Treatment, ppm	After Fluorination, ppm	After Purification, ppm	After Double Purification, ppm	The Fraction in Absorption, after Purification, ppm
C	0.0035	1,000,000	1,000,000	1,000,000	1,000,000	100
Na	0.53	93	100	19	9	0.3
Cl	33.6	52	60	320	not found	300
K	2.1	12	13	<3.2	6	<0.2
Cr	3	970	990	440	370	38
Mn	13.3	1.0	2.0	0.18	not found	<0.07
Fe	2.7	180	185	<40	5	<3
Cu	3,8	24	23	2.0	1.3	0.2
Zn	1.1	42	47	14	9	0.4
Br	6.8	0.13	0.23	2.0	1.3	0.2
Cd	2520	<0.4	<0.26	<0.35	<0.24	<25
In	197	<0.24	<0.51	<0.55	<0.15	<3
Ba	1.3	17	18	<3.4	<0.35	<0.1
Sm	5600	0.00056	0.0008	<0.00054	<0.0005	<0.1
Eu	4570	0.00017	0.00025	<0.00006	<0.0005	<0.01
Gd	48,800	<0.013	<0.013	<0.013	<0.064	<18

## Data Availability

No new data were created for this review article.

## References

[1] DeCarli P.S., Jamieson J.C. (1961). Formation of Diamond by Explosive Shock. Science.

[2] Greiner N.R., Philips D.S., Johnson J.C. (1988). Diamonds in detonation soot. Nature.

[3] Aleksenskii A.E., Baidakova M.V., Vul’ A.Y., Siklitskii V.I. (1999). The structure of diamond nanoclusters. Phys. Solid State.

[4] Danilenko V.V. (2004). On the history of the discovery of nanodiamond synthesis. Phys. Solid State.

[5] Mochalin V.N., Shenderova O., Dean H., Gogotsi Y. (2012). The properties and applications of nanodiamonds. Nat. Nanotechnol..

[6] Qin J.-X., Yang X.-G., Lv C.-F., Li Y.-Z., Liu K.-K., Zang J.-H., Yang X., Dong L., Shan C.-X. (2021). Nanodiamonds: Synthesis, properties, and applications in nanomedicine. Mater. Des..

[7] Вуль А.Я., Шендерoва О.А. (2016). Детoнациoнные Нанoалмазы. Технoлoгия, Структура, Свoйства и Применения.

[8] Dolmatov V.Y. (2001). Detonation synthesis ultradispersed diamonds: Properties and applications. Rus. Chem. Rev..

[9] Baidakova M., Vul’ A. (2007). New prospects and frontiers of nanodiamond clusters. J. Phys. D Appl. Phys..

[10] Holt K.B. (2007). Diamond at the nanoscale: Applications of diamond nanoparticles from cellular biomarkers to quantum computing. Philos. Trans. R. Soc. A.

[11] Zou Q., Li Y.G., Zou L.H., Wang M.Z. (2009). Characterization of structures and surface states of the nanodiamond synthesized by detonation. Mater. Charact..

[12] Williams O. (2011). Nanocrystalline diamond. Diam. Relat. Mater..

[13] Paci J.T., Man H.B., Saha B., Ho D., Schatz G.C. (2013). Understanding the Surfaces of Nanodiamonds. J. Phys. Chem. C.

[14] Max von Laue – Nobel Lecture Concerning the Detection of X-ray Interferences; NobelPrize.org. Nobel Prize Outreach AB 2024. https://www.nobelprize.org/prizes/physics/1914/laue/lecture/.

[15] Shull C.G. (1995). Early development of neutron scattering. Rev. Mod. Phys..

[16] Hamley I.W. (2022). Small-Angle Scattering: Theory, Instrumentation, Data, and Applications.

[17] Berne B.J., Pecora R. (2000). Dynamic Light Scattering.

[18] Krugger A., Kataoka F., Ozawa M., Fujino T., Suzuki Y., Aleksenskii A.E., Vul’ A.Y., Osawa E. (2005). Unusually tight aggregation in detonation nanodiamond: Identification and disintegration. Carbon.

[19] Aleksenskiy A.E., Eydelman E.D., Vul’ A.Y. (2011). Deagglomeration of Detonation Nanodiamonds. Nanosci. Nanotechnol. Lett..

[20] Aleksenskii A., Bleuel M., Bosak A., Chumakova A., Dideikin A., Dubois M., Korobkina E., Lychagin E., Muzychka A., Nekhaev G. (2021). Clustering of diamond nanoparticles, fluorination and efficiency of slow neutron reflectors. Nanomaterials.

[21] Nesvizhevsky V.V., Villain J. (2017). The discovery of the neutron and its consequences (1930–1940). C. R. Phys..

[22] Perrin F. (1939). Calcul relatif aux conditions éventuelles de transmutation en chaine de l’uranium. C. R..

[23] Fermi F., Zinn W.H. (1946). Reflection of neutrons on mirrors. Phys. Rev..

[24] Marguet S. (2018). Neutron Reflectors. The Physics of Nuclear Reactors.

[25] Mezei F. (1972). Neutron spin echo: A new concept in polarized thermal neutron techniques. Z. Phys..

[26] Hino M., Tasaki S., Kawabata Y., Ebisawa T., Geltenbort P., Brenner T., Butterworth J., Gaehler R., Achiwa N., Utsuro M. (2003). Development of a very cold neutron spin interferometer at the ILL. Phys. B.

[27] Nico J.S., Dewey M.S., Gilliam D.M., Wietfeldt F.E., Fei X., Snow W.M., Greene G.L., Pauwels J., Eykens R., Lamberty A. (2005). Measurement of the neutron lifetime by counting trapped protons in a cold neutron beam. Phys. Rev. C.

[28] Maruyama R. (2005). Cold and very cold neutron radiography for high contrast neutron imaging in kyoto university reactor. J. Radioanal. Nucl. Chem..

[29] Yue A.T., Dewey M.S., Gilliam D.M., Greene G.L., Laptev A.B., Nico J.S., Snow W.M., Wietfeldt F.E. (2013). Improved determination of the neutron lifetime. Phys. Rev. Lett..

[30] Piegsa F.M. (2013). New concept for a neutron electric dipole moment search using a pulsed beam. Phys. Rev. C.

[31] Oda T., Hino M., Kitaguchi M., Filter H., Geltenbort P., Kawabata Y. (2017). Towards a high-resolution TOF-MIEZE spectrometer with very cold neutrons. Nucl. Instrum. Methods Phys. Res. Sect. A.

[32] Nesvizhevsky V.V. (2022). Why very cold neutrons could be useful for neutron-antineutron oscillation searches. J. Neutron Res..

[33] Snow W.M., Haddock C., Heacock B. (2022). Searches for exotic interactions using neutrons. Symmetry.

[34] Gour S. (2010). Manufacturing Nano-Sized Powders Using Salt- and Sugar-Assisted Milling. Master’s Thesis.

[35] Lin C.R., Wei D.H., Dao M.K.B., Chung R.J., Cha Chung M.H. (2013). Chang Nanocrystalline diamond particles prepared by high-energy ball milling method. Appl. Mech. Mater..

[36] Fedoseev D.V., Varshavskaya I.G., Lavrent’ev A.V., Deryaguin B.V. (1985). Phase transformations in highly disperse powders during their rapid heating and cooling. Powder Technol..

[37] Alam M., DebRoy T., Roy R., Breval E. (1989). Diamond formation in air by the Fedoseev-Derjaguin laser process. Carbon.

[38] Baidakova M.V., Kukushkina Y.A., Sitnikova A.A., Yagovkina M.A., Kirilenko D.A., Sokolov V.V., Shestakov M.S., Vul’ A.Y., Zousman B., Levinson O. (2013). Structure of nanodiamonds prepared by laser synthesis. Phys. Solid State.

[39] Zousman B., Levinson O., Williams O. (2014). Pure nanodiamonds produced by laser-assisted technique. Nanodiamond.

[40] Ultrasonic Synthesis of Nanodiamonds. www.hielscher.com.

[41] Angus J.C., Hayman C.C. (1988). Low-pressure, metastable growth of diamond and Diamondlike phases. Science.

[42] Kharisov B.I., Kharissova O.V., Chávez-Guerrero L. (2010). Synthesis Techniques, Properties, and Applications of Nanodiamonds. Synth. React. Inorg. Met.-Org. Nano-Met. Chem..

[43] Jiang M., Chen C., Wang P., Hu X. (2022). Diamond formation mechanism in chemical vapor deposition. Proc. Natl. Acad. Sci. USA.

[44] Fermi E. (1965). A Course in Neutron Physics in Collected Papers.

[45] Fermi E., Marshall L. (1947). Interference phenomena of slow neutrons. Phys. Rev..

[46] Amaldi E., Fermi E. (1936). On the absorption and the diffusion of slow neutrons. Phys. Rev..

[47] Maier-Leibnitz H., Springer T. (1963). The use of neutron optical devices on beam-hole experiments on beam-hole experiments. J. Nucl. Energy Part A/B React. Sci. Technol..

[48] Pontecorvo B. (1941). Neutron well logging: New geological method based on nuclear physics. Oil Gas J..

[49] Feldman W.C., Maurice S., Lawrence D.J., Little R.C., Lawson S.L., Gasnault O., Wiens R.C., Barraclough B.L., Elphic R.C., Prettyman T.H. (2001). Evidence for water ice near the lunar poles. J. Geophys. Res..

[50] Bragg W.H., Bragg W.L. (1913). The reflection of X-rays by crystals. Proc. R. Soc. Lond. A.

[51] Mitchell D.P., Powers P.N. (1936). Bragg Reflection of Slow Neutrons. Phys. Rev..

[52] Fermi E. (1936). Sul moto dei neutroni nelle sostanze idrogenate. Rice Sci..

[53] Anderson H.L., Fermi E., Marshall L. (1946). Production of low energy neutrons by filtering through graphite. Phys. Rev..

[54] Egelstaff P.A., Pease R.S. (1954). The design of cold neutron filters. J. Sci. Instrum..

[55] Koester L., Rauch H., Seymann E. (1991). Neutron scattering lengths: A survey of experimental data and methods. At. Data Nucl. Dada Tables.

[56] Sears V.F. (2006). Neutron scattering lengths and cross sections. Neutr. News.

[57] Zeldovich Y.B. (1959). Storage of cold neutrons. J. Exp. Theor. Phys..

[58] Luschikov V.I., Pokotilovsky Y.N., Strelkov A.V., Shapiro F.L. (1969). Observation of ultracold neutrons. JETP Lett..

[59] Ignatovich V.K. (1990). The Physics of Ultracold Neutrons.

[60] Golub R., Richardson D., Lamoreaux S.K. (1991). Ultra-Cold Neutrons.

[61] Mezei F. (1976). Novel polarized neutron devices: Supermirror and spin component amplifier. Commun. Phys..

[62] Schanzer C., Schneider M., Boni P. (2016). Neutron optics: Towards applications for hot neutrons. J. Phys. Conf. Ser..

[63] Eder K., Gruber M., Zeilinger A., Gahler R., Mampe W., Drexel W. (1989). The new very-cold-neutron optics facility at ILL. Nucl. Instrum. Methods Phys. Res. Sect. A.

[64] Nesvizhevsky V.V., Lychagin E.V., Muzychka A.Y., Strelkov A.V., Pignol G., Protasov K.V. (2008). The reflection of very cold neutrons from diamond powder nanoparticles. Nucl. Instrum. Methods Phys. Res. Sect. A.

[65] Lychagin E.V., Muzychka A.Y., Nesvizhevsky V.V., Pignol G., Protasov K.V., Strelkov A.V. (2009). Storage of very cold neutrons in a trap with nano-structured walls. Phys. Lett. B.

[66] Nesvizhevsky V., Cubitt R., Lychagin E., Muzychka A., Nekhaev G., Pignol G., Protasov K., Strelkov A. (2010). Application of Diamond Nanoparticles in Low-Energy Neutron Physics. Materials.

[67] Cubitt R., Lychagin E.V., Muzychka A.Y., Nekhaev G.V., Nesvizhevsky V.V., Pignol G., Protasov K.V., Strelkov A.V. (2010). Quasi-specular reflection of cold neutrons from nano-dispersed media at above-critical angles. Nucl. Instrum. Methods Phys. Res. Sect. A.

[68] Klinkby E., Lauritzen B., Nonbol E., Willendrup P.K., Filges U., Wohlmuther M., Gallmeier F.X. (2013). Interfacing MCNPX and McStas for simulation of neutron transport. Nucl. Instrum. Methods Phys. Res. Sect. A.

[69] Nesvizhevsky V., Koester U., Dubois M., Batisse N., Frezet L., Bosak A., Gines L., Williams O. (2018). Fluorinated nanodiamonds as unique neutron reflector. Carbon.

[70] Nesvizhevsky V.V., Dubois M., Gutfreund P., Lychagin E.V., Nezvanov A.Y., Zhernenkov K.N. (2018). Effect of nanodiamond fluorination on the efficiency of quasispecular reflection of cold neutrons. Phys. Rev. A.

[71] Grammer K.B., Galmeier F.X. (2018). Implementation of a small-angle scattering model in MCNPX for very cold neutron reflector studies. J. Phys. Conf. Ser..

[72] Teshigawara M., Tsuchiawa Y., Ichikawa G., Takata S., Mishima K., Harada M., Ooi M., Kawamura Y., Kai T., Ohira-Kawamura S. (2019). Measurement of neutron scattering cross section of nano-diamond with particle diameter of approximately 5 nm in energy range of 0.2 meV to 100 meV. Nucl. Instrum. Methods Phys. Res. Sect. A.

[73] Bosak A., Dideikin A., Dubois M., Ivankov O., Lychagin E., Muzychka A., Nekhaev G., Nesvizhevsky V., Nezvanov A., Schweins R. (2020). Fluorination of diamond nanoparticles in slow neutron reflectors does not destroy their crystalline cores and clustering while decreasing neutron losses. Materials.

[74] Grammer K.B., Galmeier F.X. (2020). The small-angle neutron scattering extension in MCNPX and the SANS cross section for nanodiamonds. Nucl. Instrum. Methods Phys. Res. Sect. A.

[75] Jamalipour M., Zanini L., Gorini G. (2020). Implementation of Neutron Reflection with Nano-Dispersed Media in Geant4. J. Surf. Investig..

[76] Aleksenskii A., Bleuel M., Bosak A., Chumakova A., Dideikin A., Dubois M., Korobkina E., Lychagin E., Muzychka A., Nekhaev G. (2021). Effect of particle sizes on the efficiency of fluorinated nanodiamond neutron reflectors. Nanomaterials.

[77] Granada J.R., Damian J.I.M., Dawidowski J., Robledo J.I., Helman C., Romanelli G., Skoro G. (2021). Development of neutron scattering kernels for cold neutron reflector materials. J. Neutron Res..

[78] Chernyavsky S.M., Dubois M., Korobkina E., Lychagin E.V., Muzychka A.Y., Nekhaev G.V., Nesvizhevsky V.V., Nezvanov A.Y., Strelkov A.V., Zhernenkov K.N. (2022). Enhanced directional extraction of very cold neutrons using a diamond nanoparticle powder reflector. Rev. Sci. Instrum..

[79] Jamalipour M., Zanini L., Klinkby E.B., Gorini G., Willendrup P.K. (2022). Improved beam extraction at compact neutron sources using diamonds nanoparticles and supermirrors. Nucl. Instrum. Methods Phys. Res. Sect. A.

[80] Bosak A., Dubois M., Korobkina E., Lychagin E., Muzychka A., Nekhaev G., Nesvizhevsky V., Nezvanov A., Saerbeck T., Schweins R. (2023). Effect of nanodiamond sizes on the efficiency of the quasi-specular reflection of cold neutrons. Materials.

[81] Teshigawara M., Ikeda Y., Yan M.F., Muramatsu K., Sutani K., Fukuzumi M., Noda Y., Koizumi S., Saruta K., Otake Y. (2023). New Material Exploration to Enhance Neutron Intensity below Cold Neutrons: Nanosized Graphene Flower Aggregation. Nanomaterials.

[82] Rizzi N., Damain J.I.M., Kittelmann T., Lauritzen B., Klinkby E.B., Estiez Q., Santoro V. (2024). Benchmarking of the NCrystal SANS Plugin for Nanodiamonds. Nucl. Sci. Eng..

[83] DiJulio D.D., Damian J.I.M., Bernasconi M., Campi D., Gorini G., Kittelmann T., Klinkby E., Muhrer G., Ramic K., Rizzi N. (2023). Thermal scattering libraries for cold and very-cold neutron reflector materials. Eur. Phys. J. Web Conf..

[84] Aleksenski A.E., Baidakova M.V., Trofimuk A.D., Tudupova B.B., Chizhikova A.S., Svidchenko A.V. (2023). Stable hydrosol prepared by deaggregation from laser synthesis nanodiamond. Nanosyst. Phys. Chem. Math..

[85] Maleev S.V., Toperverg B.P. (1980). Low-angle multiple scattering by static inhomogeneities. JETP Lett..

[86] Steyerl A. (1974). Experiments with a neutron bottle. Z. Phys..

[87] Nesvizhevsky V.V. (2002). Interaction of neutrons with nanoparticles. Phys. At. Nucl..

[88] Arzumanov S.S., Bondarenko L.N., Geltenbort P., Morozov V.I., Panin Y.N. (2005). Cold-neutron storage owing to diffusion reflection. Phys. At. Nucl..

[89] Арзуманoв С.С., Бoндаренкo Л.Н., Гельтенбoрт П., Мoрoзoв В.И., Панин Ю.Н. (2005). Анализ Вoзмoжнoсти Применения Алмазных Нанoструктур Для Отражения и Хранения Хoлoдных Нейтрoнoв.

[90] Artem’ev V.A. (2006). Estimation of neutron reflection from nanodispersed materials. At. Energy.

[91] Lychagin E.V., Kartashov D.G., Muzychka A.Y., Nesvizhevsky V.V., Nekhaev G.V., Strelkov A.V. (2002). Mechanism of small variations in energy of ultracold neutrons interacting with a surface. Phys. At. Nucl..

[92] Ландау Л.Д., Лифшиц Е.М. (1989). Квантoвая Механика (Нерелятивистская Теoрия).

[93] Давыдoв А.С. (1973). Квантoвая Механика.

[94] Beckurts K.H., Wirts K. (1964). Neutron Physics.

[95] Ignatovich V.K., Shabalin E.P. (2007). Algebraic method for calculating a neutron albedo. Phys. At. Nucl..

[96] Vereschagin A.L., Sakovich G.V., Komarov V.F., Petrov E.A. (1994). Properties of ultrafine diamond clusters from detonation synthesis. Diam. Relat. Mater..

[97] Krylov A.R., Lychagin E.V., Muzychka A.Y., Nesvizhevsky V.V., Nekhaev G.V., Strelkov A.V., Ivanov A.S. (2011). Study of bound hydrogen in powders of diamond nanoparticles. Crystall. Rep..

[98] Piegsa F.M., Fertl M., Ivanov S.N., Kreuz M., Leung K.K.H., Schmidt-Wellenburg P., Soldner T., Zimmer O. (2014). New source for ultracold neutrons at the Institut Laue-Langevin. Phys. Rev. C.

[99] Sheng P. (1990). Scattering and Localization of Classical Waves in Random Media.

[100] Schelten J., Shmatz W.J. (1980). Multiple-scattering treatment for small-angle scattering problems. J. Appl. Cryst..

[101] Feigin L.A., Svergun D.I. (1987). Structure Analysis by Small-Angle X-ray and Neutron Scattering.

[102] Sabine T.M., Bertram W.K. (1999). The use of multiple-scattering data to enhance small-angle neutron scattering experiments. Acta Crystal..

[103] Nico J.S., Snow W.M. (2005). Fundamental neutron physics. Annu. Rev. Nucl. Part. Sci..

[104] Abele H. (2008). The neutron. Its properties and basic interactions. Prog. Part. Nucl. Phys..

[105] Ishimaru A. (1999). Wave Propagation and Scattering in Random Media.

[106] Remizovich V.S. (1984). Theoretical description of elastic reflection of particles (photons) incident at grazing angles without the use of the diffusion approximation. Sov. J. Exp. Theor. Phys..

[107] Ignatovich V.K., Nesvizhevsky V.V. (2014). Reflection of Slow Neutrons from Nanorod Powder. At. Energy.

[108] Nesvizhevsky V.V. (2011). Reflectors for VCN and applications of VCN. Rev. Mex. Fis..

[109] Mie G. (1908). Beitrage zur Optik truber Medien, speziell kolloidaler Metallosungen. Ann. Phys..

[110] Nesvizhevsky V.V., Petukhov A.K., Protasov K.V., Voronin A.Y. (2008). Centrifugal quantum states of neutrons. Phys. Rev. A.

[111] Cubitt R., Fragneto G. (2002). D17: The new reflectometer at the ILL. Appl. Phys. A.

[112] Petit T., Puskar L. (2018). FTIR spectroscopy of nanodiamonds: Methods and interpretation. Diam. Rel. Mat..

[113] Mitev D.P., Townsend A.T., Paull B., Nesterenko P.N. (2014). Screening of elemental impurities in commercial detonation nanodiamond using sector field inductively coupled plasma-mass spectrometry. J. Mater. Sci..

[114] Dolmatov V.Y. (2007). Detonation-synthesis nanodiamonds: Synthesis, structure, properties and applications. Rus. Chem. Rev..

[115] Pina-Salazar E.-Z., Urita K., Hayashi T., Futamura R., Vallejos-Burgos F., Włoch J., Kowalczyk P., Wiśniewski M., Sakai T., Moriguchi I. (2017). Water Adsorption Property of Hierarchically Nanoporous Detonation Nanodiamonds. Langmuir.

[116] Pina-Salazar E.-Z., Kukobat R., Futamura R., Hayashi T., Toshio S., Osawa E., Kaneko K. (2018). Water-selective adsorption sites on detonation nanodiamonds. Carbon.

[117] Batsanov S.S., Osavchuk A.N., Naumov S.P., Gavrilkin S.M., Leskov A.S., Mendis B.G., Beeby A., Batsanov A.S. (2018). Novel synthesis and properties of hydrogen-free detonation nanodiamond. Mater. Chem. Phys..

[118] Sears V.F. (2018). Water-selective adsorption sites on detonation nanodiamonds. Carbon.

[119] Ersez T., Osborn J.C., Lu W., Mata J.P. (2018). Small angle and inelastic scattering investigation of nanodiamonds. Phys. B.

[120] Aleksenskii A.E., Osipov V.Y., Dideikin A.T., Vul’ A.Y., Adriaenssens G.J., Afanas’ev V.V. (2000). DUltradisperse diamond cluster aggregation studied by atomic force microscopy. Tech. Phys. Lett..

[121] Aleksenskii A.E., Shvidchenko A.V., Eidel’man E.D. (2012). The applicability of dynamic light scattering to determination of nanoparticle dimensions in sols. Tech. Phys. Lett..

